# Controlling the calcium carbonate microstructure of engineered living building materials†

**DOI:** 10.1039/d1ta03990c

**Published:** 2021-10-29

**Authors:** Alexandra Clarà Saracho, Lorenzo Lucherini, Matteo Hirsch, Hannes M. Peter, Dimitrios Terzis, Esther Amstad, Lyesse Laloui

**Affiliations:** Laboratory of Soil Mechanics, Swiss Federal Institute of Technology Lausanne Switzerland alexandra.clarasaracho@epfl.ch; Soft Matter Laboratory, Swiss Federal Institute of Technology Lausanne Switzerland; Stream Biofilm and Ecosystem Research Laboratory, Swiss Federal Institute of Technology Lausanne Switzerland

## Abstract

The fabrication of responsive soft materials that enable the controlled release of microbial induced calcium carbonate (CaCO_3_) precipitation (MICP) would be highly desirable for the creation of living materials that can be used, for example, as self-healing construction materials. To obtain a tight control over the mechanical properties of these materials, needed for civil engineering applications, the amount, location, and structure of the forming minerals must be precisely tuned; this requires good control over the dynamic functionality of bacteria. Despite recent advances in the self-healing of concrete cracks and the understanding of the role of synthesis conditions on the CaCO_3_ polymorphic regulation, the degree of control over the CaCO_3_ remains insufficient to meet these requirements. We demonstrate that the amount and location of CaCO_3_ produced within a matrix, can be controlled through the concentration and location of bacteria; these parameters can be precisely tuned if bacteria are encapsulated, as we demonstrate with the soil-dwelling bacterium *Sporosarcina pasteurii* that is deposited within biocompatible alginate and carboxymethyl cellulose (CMC) hydrogels. Using a competitive ligand exchange mechanism that relies on the presence of yeast extract, we control the timing of the release of calcium ions that crosslink the alginate or CMC without compromising bacterial viability. With this novel use of hydrogel encapsulation of bacteria for on-demand release of MICP, we achieve control over the amount and structure of CaCO_3_-based composites and demonstrate that *S. pasteurii* can be stored for up to 3 months at an accessible storage temperature of 4 °C, which are two important factors that currently limit the applicability of MICP for the reinforcement of construction materials. These composites thus have the potential to sense, respond, and heal without the need for external intervention.

## Abstract 2

Biomimetic regulating technique of MICP through bacteria-laden hydrogel beads for CaCO_3_ phase control and the creation of engineered building living materials.

## Graphical abstract

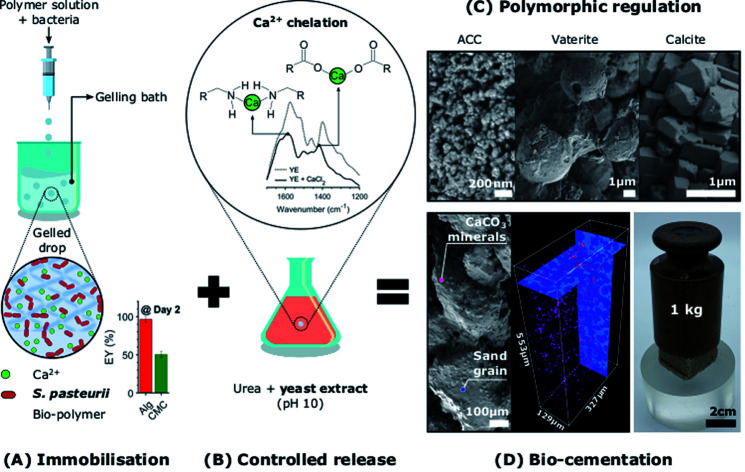

## 1 Introduction

Calcium carbonate (CaCO_3_) is one of the most abundant materials in the world, used, for example, in the cement industry,^1^ for papermaking,^2^ and drug delivery.^3,4^ It also plays an important role in nature, for example, for paleoclimate reconstructions,^5^ ocean acidification,^6^ and biomineralisation.^7–9^ CaCO_3_ has three mineral polymorphs that are in order of decreasing solubility and increasing thermodynamic stability: vaterite, aragonite, and calcite.^10^ In addition, metastable hydrated forms including monohydrocalcite, ikaite, calcium carbonate hemihydrate, and amorphous calcium carbonate (ACC) exist.^11,12^

Different CaCO_3_ polymorphs may be precipitated by bacteria under varying synthetic conditions, such as supersaturation^13–16^ and pH,^15,17–20^ through a process known as microbially induced calcium carbonate precipitation (MICP). When the concentration of calcium and carbonate ions exceeds the solubility product of the nucleating polymorph such that the solution is supersaturated, new CaCO_3_ minerals form.^10,15,21^ If the degree of supersaturation or the pH is high, calcite is precipitated *via* ACC and vaterite, while calcite forms directly at lower supersaturation levels or pH values closer to neutral conditions.^13–15,18^ During MICP, urea is hydrolysed such that the pH increases, leading to an increased amount of carbonate ions (CO_3_^2−^), and hence an increased degree of supersaturation. Once CaCO_3_ precipitates, the pH is reduced, resulting in a lower degree of supersaturation. Owing to the limited control over the rate of urea hydrolysis, and hence supersaturation, this precipitation pathway hampers structural and compositional control over the resulting CaCO_3_ minerals.

Yet, this control would be essential because calcite has a higher consolidation capacity than other metastable phases of CaCO_3_, such as ACC and vaterite,^22,23^ thereby resulting in denser materials that are stiffer. Despite the limited control over the polymorph formation, MICP has been successfully exploited to produce CaCO_3_ minerals for soil reinforcement,^24–28^ concrete crack repair,^29,30^ carbon capture and storage,^31^ and bone tissue engineering applications.^32,33^ To increase the CaCO_3_ production rate, CaCO_3_ precipitating bacteria have been introduced in soils as liquid suspensions of vegetative or freeze–dried cells. This was achieved through injection^34,35^ or surface percolation,^25^ pre-mixing,^36^ spraying,^37^ or permeation under electric fields.^38^ However, the short shelf-life and lack of spatial control of these delivery strategies have hindered their commercial use. A more widespread application of MICP requires a better control over the location of bacteria and their metabolism to be optionally activated when required. To leverage the full potential of this method, the spatio-temporal regulation of bacteria must be coupled with a tight control over the structure of the resulting CaCO_3_ minerals.

Concrete can be self-healing if, for example, it contains bacteria that are deposited at well-defined locations.^39–41^ This positional control can be achieved by encapsulating bacteria in clay particle,^39^ melamine^40^ or aerated concrete granules^41^ and releasing them through mechanical breakage of the capsule upon concrete cracking, thereby initiating the healing of these cracks. Owing to the low tensile strength of concrete, cracking is widely and commonly encountered; this makes such an autonomous healing upon cracking useful. By contrast, saturated cohesionless soils, like sand, have no tensile strength, such that damage by cracking does not occur. Alternative bacteria release mechanisms that make cohesionless soils responsive to environmental and mechanical damage remain to be established.

Several strategies to immobilise bacteria while maintaining their metabolic activity have been used in environmental applications, such as adsorption on surfaces, cell crosslinking, encapsulation, and entrapment.^42^ Hydrogels offer an ideal living environment for bacteria as they contain a high water content, are non-toxic and biodegradable, such that no additional carbon is returned to the biosphere.^42^ To enable a controlled bacteria release however, hydrogels must be responsive. Certain hydrogels readily respond to external stimuli, including light, enzymes, temperature changes, proteins, and pore pressure variations.^43–45^ For example, Ca^2+^-crossliked alginate (Alg) displays pH-dependent mechanical properties.^46,47^ Unfortunately, Ca^2+^ is only released from alginate if the pH is reduced below 5, which compromises bacterial viability.^48^ Despite the envisioned advantages of embedding bacteria in hydrogel beads, the controlled release that is coupled with the recognition of specific MICP components and that does not compromise bacterial viability has not yet been demonstrated.

**Fig. 1:**
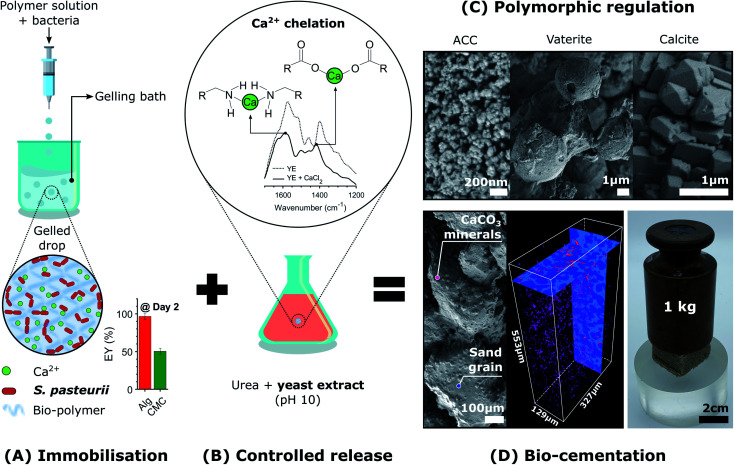
Schematic illustration of the biomimetic regulating technique of MICP for CaCO_3_ phase control and the creation of functional living materials. (A) *Sporosarcina pasteurii* bacteria are loaded within hydrogel microparticles *via* extrusion dripping and Ca^2+^ crosslinking. (B) To trigger the release of the calcium ions from the hydrogel we exploit competitive ligand exchanges between the hydrogel and the peptides in the yeast extract. Bacteria released from the hydrogel initiate the precipitation of CaCO_3_ through urease catalysed hydrolysis. The encapsulation of MICP leads to (C) CaCO_3_ crystals with tailorable microstructures and (D) the creation of living and responsive geo-materials capable of binding soil particles *in situ*.

Here, we introduce the use of a competitive displacement strategy to control the location and timing of the MICP reaction, and the structure and properties of the resulting CaCO_3_ minerals while preserving bacterial viability. This is achieved by loading *Sporosarcina pasteurii* bacteria into alginate and carboxymethyl cellulose (CMC) hydrogel beads and using these beads to control the concentration of bacteria, as shown in Fig. 1A. To tune the amount and structure of the forming CaCO_3_, we exploit a competitive ligand exchange that controls the growth and metabolic activity of bacterial cells,^41^ and offers an external control over the timing of the CaCO_3_ formation (Fig. 1B). The precipitation of CaCO_3_ decreases the pH, thereby initiating a competition for the Ca^2+^ binding between alginate and the peptides present in the yeast extract. The resulting synergistic effect of the calcium-binding ligands on supersaturation is used to control the phase, morphology, and yield of the precipitated CaCO_3_ (Fig. 1C), thereby technically supporting implementation in many processes and applications. We focus on showcasing this approach to produce site-specific biomineralised composites that bind soil particles together by embedding beads in sand specimens due to contemporary interest in the use of MICP for soil reinforcement applications (Fig. 1D).^25,27,49^ However, as a novel biomimetic regulating technique of MICP, this platform technology opens new possibilities for the design of CaCO_3_ crystals with tailorable microstructures, and hence, mechanical properties. In addition, it enables the design of living materials that endow geosystems with the ability to sense, heal, and develop immunity to harmful environmental, climatic, and human actions, vital for the development of sustainable practices.

## 2 Results and discussion

### 2.1 Immobilisation of *Sporosarcina pasteurii* in the hydrogel beads

**Fig. 2:**
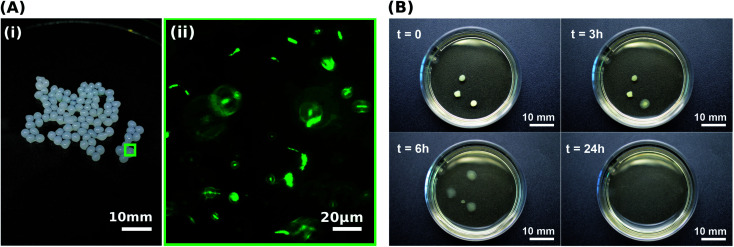
Bacterial viability after immobilisation in the alginate-based hydrogel beads and gel liquefaction evaluation over time. (A) Photograph of alginate-based hydrogel beads (i) and representative live staining fluorescent image of *S. pasteurii* immobilised in an alginate-based hydrogel bead (ii). Live bacteria are labelled in green. (B) Time-lapse images of the liquefaction of the alginate-based hydrogel beads in 1.0 M YU medium (pH 10) triggering the release of immobilised *S. pasteurii* over a 24 hour period.

To control the spatial distribution and concentration of bacteria, we immobilise *Sporosarcina pasteurii* within alginate and CMC hydrogels *via* extrusion dripping. A polymer solution containing a suspension of bacteria is pumped through a needle and dripped into a solidifying Ca^2+^-containing aqueous bath using a volume-controlled syringe pump. The diameter of the forming solid microparticles is controlled with the tip diameter, as shown in Fig. 1A. The distance between the needle tip and the gelling bath was empirically adjusted to minimise coalescence between two consecutive drops before gelling, thus reducing the fraction of tear-shaped beads,^50^ whilst ensuring a compact setup to fit within a laminar flow hood. The process parameters used in this study yield a production rate of 100 beads per minute, with a mean bead diameter of ∼1 mm and ∼0.75 mm for alginate and CMC, respectively, as shown in Fig. 2A(i) and summarised in Table S1.† To ensure that enough urea is hydrolysed at a given location, and hence that enough CaCO_3_ is formed, we adjust the number of bacteria per bead to 4.4 × 10^7^ cfu per bead. To assess the mechanical properties of the capsules, we performed compression tests on them. Stress–strain curves of alginate reveal a breaking stress of 31.5 ± 9.7 kPa and a fracture strain of 68 ± 4.7% (Fig. S1†). The compressive modulus, calculated as the slope of the initial linear region ranging between 5–15% strain, was 12.1 ± 0.4 kPa (Table S2†).

A prerequisite for bacteria to be embedded in hydrogel beads is the retention of bacterial survival and metabolic activity after the manufacturing process. Live staining fluorescent imaging of *S. pasteurii* immobilised in alginate-based hydrogel beads (Fig. 2A(ii)) demonstrates that bacteria in a hydrogel matrix can be extruded in the form of beads while providing the environment to keep them live and functional. To compare the viability of immobilised bacteria to that of mobile ones for a 1 week period, we incubate freeze–dried bacteria in a yeast extract–urea (YU) medium (pH 10) for a 24 hour period and subsequently transfer them into an ammonium–yeast extract (NH_4_–YE) medium (ATCC 1376). Bacteria that were immobilised in alginate started to grow within 24 h, as shown by the significant increase in optical density measured at 600 nm (OD_600_) of above 4 from 0 to 1 day in Fig. S2A.† Within day 2, the encapsulation yield (EY) was 96.4 ± 1.5%, which is in good agreement with previous studies of bacteria embedded in alginate reporting an EY ranging between ∼50–94%.^29,51–53^

The survival and metabolic activity of bacteria after the extrusion process is strongly influenced by the rheological properties of the hydrogel.^54^ To test if this is also the case for the system introduced here, we embed bacteria in CMC hydrogels. To increase the calcium-binding affinity of CMC, and hence the mechanical integrity of the hydrogel, we use high molecular weight CMC (250 kDa) comprised of a high content of calcium-binding carboxymethyl groups.^54^ However, the higher molecular weight gives the pre-gelled solution a viscosity that is 40-fold higher than that of alginate. Hence, the shear force imposed on the bacteria contained in this solution during the mixing and extrusion process is much higher,^54^ despite of its higher yield strain, as shown in Fig. S3.† The higher shear force required to mix the CMC solution is likely responsible for bacteria immobilised in CMC displaying a lag phase of 1 day and a slower growth rate during the first 24 h of the exponential phase than those contained in alginate, as shown in Fig. S2A.† Within day 2, the EY was 50.4 ± 3.91, in agreement with the ∼55% reported in a previous study.^55^ Remarkably, this is about 50% of the value measured for alginate. The reduction in bacterial viability was further supported by the lower urease activity in CMC (Fig. S2B†). Therefore, we attribute the enhanced bacterial viability achieved by embedding bacteria in alginate hydrogels to the lower viscosity of the pre-gelled solution, dependent on the molecular weight of the polymer, as expected from a previous study using modified molecular weight alginate to form gels for cell encapsulation.^54^ These results suggest that for the systems we study here, the viability of bacteria loaded into hydrogels is highly sensitive to the molecular weight of the pre-gelled polymer, which in turn affects the stiffness of the gelled solution.

### 2.2 Peptide-controlled release of calcium ions

To trigger the release of Ca^2+^ from alginate-based hydrogels without compromising the viability of bacteria contained in these gels, we exploit competitive ligand exchanges to liquefy the gel. If ligands possessing a higher affinity for Ca^2+^ than alginate (p*K*_a_ ∼ 4 (ref. 56)) are added to the gel, Ca^2+^ is removed from the hydrogel, thereby resulting in its liquefaction.^48^ Following a similar principle, calcium-binding proteins and peptides, such as glutamate and aspartate, which exhibit a pH-depending ion affinity, can be used to harvest calcium from alginate gels.^48^ Note that these are key amino acids present in the peptides of yeast extract, such that their pH-dependent calcium-binding affinity can be exploited to harvest Ca^2+^ ions that crosslink alginate, and hence, trigger the release of immobilised bacteria.

To test this concept, we chelated Ca^2+^ with the calcium-binding yeast extract (pH 10), and monitored the main binding sites of calcium within the peptides using Fourier-transform infrared spectroscopy (FTIR) (Fig. 1B).^57–60^ A yeast extract solution buffered at pH 10 promotes the deprotonation of the carboxylic and ammine groups in the peptides of yeast extract, which have a stronger calcium binding affinity than their protonated form.^61^ Moreover, the growth of *S. pasteurii* is strongly affected by pH, and increases with increasing pH in the range of 5.0–9.0, as shown in Fig. S4.† Thus, a pH of 10 ensures a strong calcium binding affinity of the deprotonated amino acids, while providing suitable conditions for bacterial growth. FTIR results for the dried yeast extract are in good agreement with the absorption bands measured for yeast extract derived from *Saccharomyces cerevisiae*.^62^ We assign the peaks at 1458 cm^−1^ and those in the range of 2990–2820 cm^−1^ to bending vibrations, and symmetric and asymmetric stretching vibrations of CH_2_ and CH_3_ in lipids and proteins, respectively, 1574 cm^−1^ to the amide II N–H and C–N vibrations of the peptide bond, and 1398 cm^−1^ to the C=O belonging to the COO^−^ symmetric stretching in proteins (Fig. S5A†). The addition of calcium that results in the formation of the peptide-calcium chelates causes a shift in the vibrational stretching of N–H to a higher wavenumber (3286 cm^−1^), indicating that Ca^2+^ was coordinated by the nitrogen of the amino group. A binding also occurred between Ca^2+^ and the carboxylate groups to form a –COO–Ca complex, as indicated by the decrease in intensity and shift in wavenumber to 1417 cm^−1^ of the C=O peak.^57,60^ These interactions increased the turbidity of the solution because the peptides started to flocculate after chelation with calcium (Fig. S5B†). In agreement with the optimised structures obtained from density functional theory for aspartate and glutamate,^61^ these results clearly demonstrate that the nitrogen atom and the carboxylate oxygen were involved in the chelation of Ca^2+^, as shown in Fig. 1B.

**Fig. 3:**
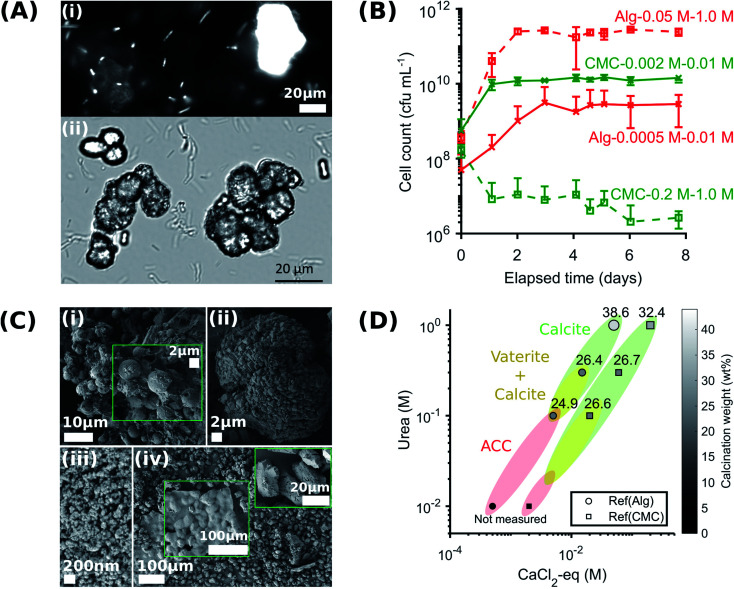
Viability of *S. pasteurii* after release from the hydrogels, and structure and yield of CaCO_3_ formed in the absence of hydrogels. (A) Observations by epifluorescence (i) and brightfield (ii) optical microscopy of bacteria and CaCO_3_ precipitation in their microenvironment, respectively; (B) flow cytometry measurements of bacteria release from the hydrogel; (C) SEM-SE2 images of vaterite spherulites in Ref(Alg)-0.015 M–0.3 M (i) and amorphous calcium carbonate nano-aggregates in Ref(CMC)-0.002 M–0.01 M (iii), and calcite with rhombohedral morphology in Ref(Alg)-0.05 M–1.0 M (ii) and Ref(CMC)-0.2 M–1.0 M (iv); and (D) CaCO_3_ phase diagram and yield (the symbol size and colour are proportional to the calcination weight loss).

A key factor that determines the efficiency of the system in producing CaCO_3_ is the bacterial viability and proliferation after they have been released from the hydrogel into the YU medium (pH 10), which occurred over a 24 hour period (Fig. 2B). To quantify this parameter, we precipitated CaCO_3_*in vitro* in a solution containing 0.01–1 M urea and 0.0005–0.2 M CaCl_2_-eq. at room temperature, corresponding to typical solution concentrations used for the reinforcement of construction materials.^25,34^ The adopted sample identification notation refers to the hydrogel, and the CaCl_2_-eq. and urea concentrations used, as detailed in Fig. S6.† For example, Alg-0.05 M-1.0 M refers to bacteria immobilised in alginate beads, and released in a 0.05 M CaCl_2_-eq. and 1.0 M urea solution. Observations by brightfield and epifluorescence optical microscopy confirmed the viability of *S. pasteurii*, as indicated by the precipitation of CaCO_3_ minerals in their micro-environment resulting from the hydrolysis of urea into carbonate ions induced by the metabolic activity of *S. pasteurii* (Fig. 3A(i) and (ii)). Flow cytometry measurements demonstrate a rapid release of *S. pasteurii* from alginate (Fig. 3B). The release count from Alg-0.0005 M-0.01 M was 5.0 × 10^7^ cfu mL^−1^ after 2 h, increased to 3.1 × 10^9^ cfu mL^−1^ at day 3, and plateaued at 3.1 × 10^9^ cfu mL^−1^ thereafter. We assign this fast release to the increased pore sizes of the hydrogel upon removal of the crosslinking Ca^2+^. Even faster release rates were observed for Alg-0.05 M-1.0 M, where the maximum release degree of 2.5 × 10^11^ cfu mL^−1^ was measured already after day 2. We assign the faster peak in release to the higher concentration of urea (1.0 M), prompting a faster reactivation and ureolytic activity of the bacteria, as evidenced from the higher electrical conductivity measurements in Fig. S7.† Note that the measured maximum bacterial cell count was never below the number of immobilised cells, equal to 2 × 10^7^ cfu mL^−1^ and 2 × 10^9^ cfu mL^−1^ for the low and high CaCl_2_-eq. concentrations, respectively. These results further confirm that bacteria continued to grow up to 100-fold in the YU medium.

The absolute bacterial viability after release from CMC depends on the initial mass of crosslinked beads in solution, and hence on the polymer content and crosslinker concentration. The calcium content of CMC beads is four times larger than that of alginate beads (Table S3†). Nevertheless, we kept the total mass of crosslinked CMC beads equal to that of alginate, to maintain the bacterial concentration equal. The growth of bacteria saturated at 10^10^ cfu mL^−1^ in CMC-0.002 M–0.01 M. This value is similar to that observed for alginate. By contrast, the bacterial release profile from CMC-0.2 M–1.0 M decreased with time. This difference in viability may be a result of the high solution viscosity of CMC upon removal of the crosslinking Ca^2+^. The high viscosity made the separation of immobilised bacteria difficult,^54,55,63^ thereby preventing an accurate quantification of released *S. pasteurii*.

### 2.3 Structure and yield of CaCO_3_ formed in the absence of hydrogels

An important feature of any MICP system is the morphology, phase, and properties of the CaCO_3_ produced, because these parameters influence the mechanical properties of the composite. To test the influence of reactant concentration on CaCO_3_ yield, we vary the CaCl_2_ and urea concentrations in solution, in the absence of any hydrogel, keeping the relative molarities constant. The adopted sample identification notation refers to the mobile system corresponding to an immobilised counterpart. For example, (Ref)Alg-0.05 M-1.0 M refers to mobile bacteria suspended in a 0.05 M CaCl_2_-eq. and 1.0 M urea solution, matching the concentrations used for Alg-0.05 M-1.0 M. We determine the weight of precipitated CaCO_3_, using thermogravimetry analyses (TGA). We observe a main weight loss in the range of 600–800 °C, indicative of the calcination of calcium carbonate, as shown in Table S8.† The peak decomposition temperature increased with the amount of urea and CaCl_2_-eq. contained in the sample (see zoomed in plots in Table S8†). Similarly, the weight loss, and hence the CaCO_3_ precipitation yield, increases with increasing urea and CaCl_2_-eq., reaching a maximum value of 38.6 ± 3.7 wt% and 32.4 ± 8.7 for Ref(Alg)-0.05 M–1.0 M (Table S8A†) and Ref(CMC)-0.2 M–1.0 M (Table S8B†), respectively. These values are close to the 44 wt% corresponding to the stoichiometric CO_2_ amount in CaCO_3_.

The urea and CaCl_2_-eq. concentrations also influence the structure of the forming CaCO_3_, as revealed by X-ray diffraction (XRD). Samples Ref(Alg)-0.005 M–0.1 M, Ref(Alg)-0.015 M–0.3 M, and Ref(CMC)-0.02 M–0.1 M contained a mixture of calcite and vaterite, as shown in Fig. S9.† Note that the relative abundance of vaterite to calcite decreased with increasing amount of CaCl_2_-eq. and urea in the sample. These results were confirmed with IR where both ν_4_ bands at 712 and 744 cm^−1^, characteristic of crystalline vaterite and calcite respectively, were observed (Fig. S10†). Importantly, the increasing abundance of vaterite measured in the sample was accompanied by the broadening of the IR ν_3_ band at 1409 cm^−1^, and the appearance of: the ν_1_ absorbance band at 1075 cm^−1^, the broad band centred at 3260 cm^−1^ assigned to hydrogen bonded O–H stretching and the sharper band at 1645 cm^−1^ due to O–H bending (Table S4†). The last two features correspond to molecular water and are typical of hydrated calcium carbonate.^64,65^ Their presence suggest that a significant part of the CaCO_3_ contained in Ref(Alg)-0.005 M–0.1 M is amorphous calcium carbonate (ACC). This suggestion was confirmed by the splitting of the ν_3_ band at 1409 and 1466 cm^−1^ and the broad hump at ∼700–750 cm^−1^ (see zoomed in plot Fig. S10†).^15,66^ SEM images confirmed that ACC particles were also present in the samples prepared at the lowest urea concentrations (0.01 M), as shown in Fig. 3C(iii). By contrast, the samples produced with the highest urea concentration (1.0 M) only contained calcite, as confirmed by XRD and IR, where only the ν_4_ band at 744 cm^−1^ was seen (Fig. S9 and S10†).

Interestingly, the concentrations of urea and CaCl_2_-eq. also influenced the crystal morphology: vaterite contained in Ref(Alg)-0.015 M–0.3 M was present as micron-sized spherulites (Fig. 3C(i)), whereas the characteristic calcite rhombohedra arranged into mesocrystals in Ref(Alg)-0.05 M–1.0 M (Fig. 3D(ii)). The morphology of these crystals differed substantially from that of the calcite crystals in Ref(CMC)-0.2 M–1.0 M, which displayed a clear selective expression of the prismatic (110) side faces that are parallel to the *c*-axis (Fig. 3C(iv)). This crystal elongation was supported by the intensity ratio of *I*_104_/*I*_110_ obtained from the XRD patterns, which decreased from 2.0 to 1.75.

In summary, calcite formed under highest urea and CaCl_2_-eq. concentrations, while the amorphous phase and vaterite were dominant when CaCO_3_ formed at lower reagent concentrations. This phase change was accompanied by a decrease in the CaCO_3_ product yield, which was as high as 38.6 ± 3.7 wt% for Ref(Alg)-0.05 M–1.0 M and decreases to 24.9 ± 10.1 wt% for Ref(Alg)-0.005 M–0.1 M (Fig. 3D). Such a bacteria and reagent concentration-dependent polymorphic sequence, ACC → vaterite → calcite, is in agreement with a previous study conducted under various initial *S. pasteurii* concentrations and degrees of supersaturation.^16^ As a result, ACC and vaterite may be far more common than would be anticipated in abiotic systems. Considering that different CaCO_3_ mineral polymorphs impart different mechanical properties and that only calcite provides the high mechanical stability needed to form viable scaffolds,^22,23^ polymorphic regulation is highly desirable. Our results demonstrate that this can, to some extent, be done by closely tuning the bacteria and Ca^2+^ concentrations.

### 2.4 Effect of the hydrogel on the CaCO_3_ structure and yield

**Fig. 4:**
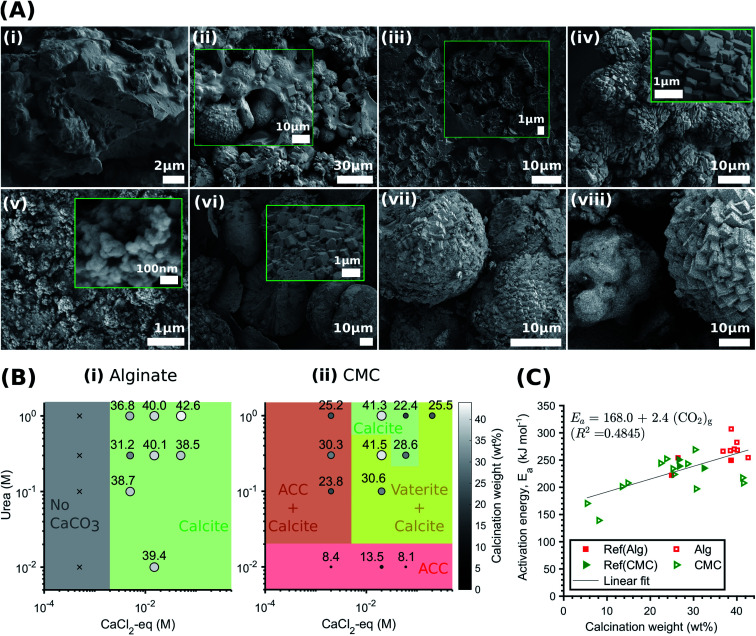
Effect of the hydrogel on the CaCO_3_ structure and yield. (A) SEM-SE2 images showing the role of urea concentration on the morphology of the calcite formed within alginate, evolving from small traces of crystalline units enmeshed in a gel-like matrix to the rhombohedral morphology of calcite: Alg-0.0005 M-0.01 M (i), Alg-0.005 M-0.1 M (ii), Alg-0.005 M-0.1 M (iii), Alg-0.05 M-1.0 M (iv). By contrast, CMC has no effect on the morphology and the CaCO_3_ formed: CMC-0.002 M–0.01 M (v), CMC-0.02 M–0.1 M (vi), CMC-0.06 M–0.3 M (vii), CMC-0.2 M–0.1 M (viii); (B) phase diagram and yield of CaCO_3_ formed within alginate (i) and CMC hydrogels (ii) (the symbol size and colour are proportional to the calcination weight loss); (C) coats-Redfern kinetics model of CaCO_3_ decomposition (*α* = 0.15–0.78 (ref. 67)).

Certain hydrogels have been shown to influence the structure and yield of the CaCO_3_ that forms within them.^33,68^ To examine if this is also the case in the systems we study here, we precipitate CaCO_3_ within alginate and CMC hydrogels and systematically vary the concentrations of urea and CaCl_2_-eq. We characterise the structure of the formed minerals with XRD and FTIR, and their amount using TGA. Remarkably, in all alginate samples except Alg-0.0005 M-0.01 M, we only detect calcite, as shown by the IR peaks corresponding to the carbonate group at 1409 cm^−1^ (ν_3_), 871 cm^−1^, and 712 cm^−1^ (ν_4_) (Fig. S11 and Table S5†).^69^ The presence of only calcite is further confirmed by the SEM images in Fig. 4B(i–iv) and the XRD patterns in Fig. S12.† These results are in stark contrast to those obtained from non-immobilised systems where ACC, vaterite, and calcite form. We attribute this discrepancy to the intermolecular interactions between the Ca^2+^ and the alginate chains in the polymer^33,68,70^ arising from the CaCO_3_ precipitation process that regulate supersaturation. During MICP, there is a rapid increase in pH due to urea hydrolysis, followed by a pH decrease due to the precipitation of CaCO_3_.^20^ With decreasing pH, the calcium-binding affinity of peptides also decreases,^61^ weakening the bonds between the Ca–oxygen and the Ca–nitrogen within the peptides that have not been precipitated into CaCO_3_. This results in enhanced competitive Ca^2+^ exchanges between alginate and the peptides until an equilibrium is approached. Because the peptides–Ca^2+^ interactions strengthen with increasing pH, we expect a faster initial CaCO_3_ precipitation rate with increasing urea concentrations, caused by a larger availability of CO_3_^2−^ (Fig. S7†). Indeed, we observe a narrowing of the O–H stretch band in the IR range of 3000–3600 cm^−1^ with decreasing urea concentrations, which arises from the formation of hydrogen bonds between the hydroxyl groups (–OH) of the alginate and calcium (see zoomed in plot in Fig. S11†).^70^ This interaction is strengthened with increasing concentrations of free calcium ions.

The pH-dependent competition for Ca^2+^ between alginate and the CaCO_3_ precipitation strongly influences the phase, morphology and yield of CaCO_3_. Once crystals nucleate and the pH decreases, the excess Ca^2+^ bound by the alginate leads to a reduction in solution supersaturation. This is likely to favour calcite precipitation,^71^ as ACC and vaterite have been shown to preferably crystallise at high supersaturation levels.^13,14^ Additionally, the gelation of alginate limits the availability of Ca^2+^ for crystal growth. If we keep the CaCl_2_-eq. constant, this explains the reduction in calcite yield with decreasing urea concentrations observed in Fig. 4E(i). Finally, it is apparent from the SEM images shown in Fig. 4A(i–iv) that the morphology evolves from small traces of crystalline units enmeshed in a gel-like matrix (Alg-0.0005 M-0.01 M) to the rhombohedral morphology of calcite (Alg-0.05 M-1.0 M), providing visual demonstration that the Ca–alginate crosslinking decreases with increasing urea concentrations. This is further supported by TGA (Fig. S13†), showing a decrease in weight loss up to 500 °C with increasing urea concentrations, attributed to the decomposition of alginate (Fig. S13A†). These results are in stark contrast to those of the CMC analogues, summarised in Fig. 4B, where the calcium-binding affinity of CMC in relation to peptides is much lower. As a result, the pH-dependent ligand competition described above is insufficient to affect the supersaturation, and hence, the CaCO_3_ precipitation process (*cf.*Fig. 3D). As a result, the urea concentration does not influence the structure or yield of CaCO_3_ precipitated in CMC.

#### 2.4.1 Calcination kinetics

The stoichiometry of CaCO_3_ determines its thermal stability, such that the relative crystallinity of the formed minerals can be evaluated using TGA.^33^ The activation energies *E*_a_ associated with the calcination of the different CaCO_3_ precipitates (*i.e.* amorphous plus crystalline phases) were calculated from TGA data using eqn (1) (Fig. S14 and S15†).^67^ Here, α (as defined in eqn (2)) is the fraction of CaCO_3_ decomposed at time *t*, *A* is a pre-exponential factor and *f*(*α*) describes the reaction model, which for the decomposition of carbonates can be expressed as 1 − *α*.1
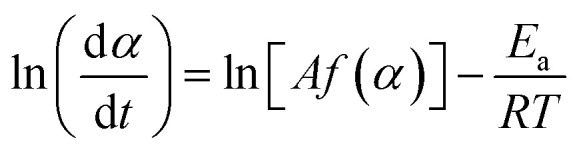
2
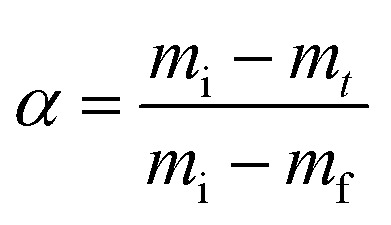
where *m*_i_ and *m*_f_ are the initial and final masses, respectively, and *m*_*t*_ is the mass at time *t* or temperature *T*, and *R* is the gas constant (8.314 J mol^−1^ K^−1^). The activation energy for CaCO_3_ upon calcination increases with calcination weight loss, and thus precipitated CaCO_3_ mass, reaching a value of 307.2 ± 29.8 kJ mol^−1^ in sample Alg-0.005 M-0.3 M, as shown in Fig. 4C (data summarised in Tables S7 and S8†). This value is higher than the average activation energy for calcification of non-immobilised controls which, for both hydrogel-free systems, is 242 kJ mol^−1^ (solid markers in Fig. 4F). Therefore, alginate plays an important role in controlling the crystallinity of the CaCO_3_ formed. Because calcination weight loss increases with CaCO_3_ polymorph stability (*cf.*Fig. 3D and 4C), our results are a strong indication that stoichiometric calcite with higher crystallinity is formed within alginate hydrogels. By contrast, *E*_a_ values for the calcination of CaCO_3_ formed within CMC had a wider distribution, which was as high as 269 kJ mol^−1^ and decreased to 139 kJ mol^−1^ in samples CMC-0.002 M–0.3 M and CMC-0.06 M–0.01 M, respectively. Note that the minimum *E*_a_ measured is close to that reported for the calcination reaction enthalpy: Δ*H* = 177.8 kJ mol^−1^.^72^ We assign this discrepancy to the different mineral phases found in varying abundances in CMC samples, as schematically shown in Fig. 4B(ii).

### 2.5 Capsule-based MICP for living building materials

To demonstrate the potential of immobilised *S. pasteurii* to produce a natural cementing element for soil reinforcement applications, we immobilised the bacteria within an alginate matrix. Non-toxic, natural polymers protect the bacteria from mechanical abrasion during transport and application while allowing for their controlled release and subsequent attachment to the soil matrix.^42^ The hydrogel matrix offers an additional benefit: facilitated storage and transportation. Extreme cold temperatures (over the range of −20 and −80 °C) necessary for stable storage of freeze–dried bacteria can pose severe limitations for deployment.^73^ Moreover, frozen bacteria must be reactivated and introduced into the soil before their metabolic activity decreases or the enzyme is degraded; these factors are sensitive to various stresses in the soil environment.^74–76^

These shortcomings can be overcome if *S. pasteurii* are immobilised within hydrogels because freeze–dried bacteria have a prolonged lifetime when embedded in these matrices. To test their stability during storage in the fridge at 4 °C, we studied bacterial viability in hydrated beads over a period of 4 months and compared it to that of freshly reactivated mobile cells. Indeed, we observe an enhanced initial viability of bacteria contained in hydrogels if stored for up to 3 months at 4 °C, as shown by the higher initial urease activity values in Fig. S16A.† These results indicate that immobilised bacteria are not subjected to detrimental osmotic shocks that could cause an influx of water, a fact that may protect them during wetting–drying cycles.^75,77^ The growth of immobilised bacteria did not significantly change over 2 months of storage at 4 °C, with a maximum OD_600_ of 6 attained at day 2 (Fig. S16B†). After 3 months of storage, the encapsulation yield (EY) decreased from 83.2 ± 5.2% to 55.5 ± 5.2% at day 2, and no growth was measured after 4 months (see zoomed in plot in Fig. S16B†). Non-immobilised fresh bacteria showed a lag phase of 1 day followed by a faster growth to an OD_600_ of 6.3 ± 0.055 at day 2. However, the difference in the initial growth rate of fresh and immobilised bacteria decreases with time and no significant difference was measured after 7 days, as shown in Fig. S16B.† Therefore, the bacterial growth and decay profile remains unaffected for up to 3 months of storage, which is in good agreement with a previous study showing that freeze–dried bacteria pre-treated with cryoprotectants and stored at 4 °C suffer a loss in viability precluding their use after 3 months.^73^

**Fig. 5:**
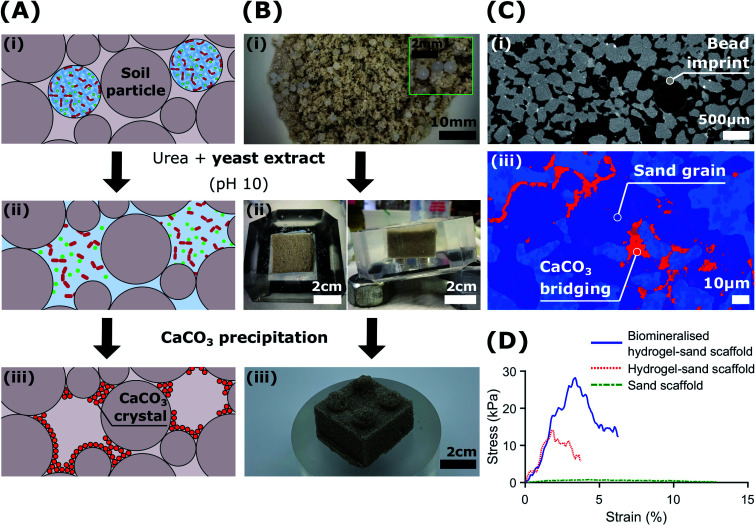
*Sporosarcina pasteurii* embedded within alginate beads for the biocementation of soil: (A) schematic illustration of capsule-based MICP in soils. Bacteria embedded in hydrogels beads are introduced in the soil and remain dormant until MICP treatment is required (i). Upon contact with the YU medium (pH 10), the calcium ions crosslinking the hydrogel are bound by the peptides of the yeast extract. This leads to the continuous release of *S. pasteurii* (ii), and the *in situ* formation of CaCO_3_ (iii). (B) By mixing bacteria embedded in hydrogel beads with the soil (i), the MICP process transforms cohesionless sand (ii) into a living building geo-material with structural integrity (iii). (C) XRCT two-dimensional slices of MICP-treated sand showing dissolved alginate bead imprints (i) and CaCO_3_ minerals binding soil particles *in situ* (light blue; pores; dark blue, sand grains; red, CaCO_3_ minerals), respectively (ii). (D) Compression tests of biomineralised hydrogel-sand scaffolds compared to those of hydrogel-sand and sand scaffolds.

To demonstrate the potential of biologic self-healing soil, we packed a mixture of bacteria-loaded alginate beads and sand in a 3D-shaped silicone mould (Table S9†), and added YU medium for 48 h to control the release of bacteria, as shown in Fig. 5A, B(i and ii). To precipitate CaCO_3_*in situ*, we cured the samples in a calcium-containing cementation solution for 10 days. During this incubation time, we formed 0.51 wt% calcite, as was determined from the X-ray μ-CT data (Table S10†). This result is in reasonable agreement with the 1.07 ± 0.32 wt% directly measured from thermogravimetric analyses (Fig. S17 and Table S11†). By forming CaCO_3_ at the soil particle constrictions (Fig. 5A(iii)), the sand gains structural integrity (Fig. 5B(iii)) and becomes load-bearing, as evinced by its ability to sustain a 1 kg in Fig. 1D. Indeed, the two-dimensional (2D) cross-sections and three-dimensional (3D) volume reconstructions confirmed the formation of a well-distributed network of bacterial CaCO_3_ minerals (red) bridging the soil grains (dark blue), as shown in Fig. 5C and S19.†

The morphology of the CaCO_3_ bonds was strongly influenced by the presence of the hydrogel matrix, as revealed by SEM images (Fig. S18†). Similar morphologies have been recently reported for cyanobacteria biomineralised hydrogel-sand scaffolds,^78^ and demonstrate potential for the engineering of mineralised hydrogels mimicking organic/inorganic composite materials in nature.^33,79–82^ As we dissolved the hydrogel beads upon contact with the YU medium and precipitated CaCO_3_, the porosity of the resulting bio-geo-material decreased from 41.5% to 27%, indicating that 14.5% of the pore space was occupied by the alginate matrix (Table S10†). Indeed, these values constitute an upper bound as drying of the biomineralised hydrogel-sand scaffolds may have caused particle rearrangement due to the shrinkage of the hydrogel. Nonetheless, the impact of hydrogel shrinkage below 100 °C is minimal, as shown by the weight loss of less than 1% in Fig. S17.†

To test if our biomineralised hydrogel-sand scaffold is sufficiently robust to sustain more demanding loads, we perform compression measurements and compare the results with those of hydrogel-sand and sand scaffolds. The compressive modulus of the biomineralised hydrogel-sand scaffold is 25 fold higher than that of sand, only exhibiting an apparent cohesion attributed to the surface tension of the moisture film surrounding each particle. The uniaxial compressive strength (UCS) increases even more: it reaches 28 kPa at 3.5% strain which is two times higher than that of the hydrogel-sand scaffold and 35 fold higher than that of sand alone, as shown in Fig. 5D. To put our results into perspective, we note that CaCO_3_ contents in the range of 0.5–2 wt% are generally required for soil erosion and liquefaction mitigation,^25,50^ 2–6% yielding UCS values in the range of 0.1–2 MPa to create load-bearing materials,^35,83–85^ and contents above 6% for bio-clogging applications requiring a notable reduction in porosity.^84,86^ However, because our samples are fabricated from biomineralised hydrogel-sand scaffolds, we can independently optimise the amount of CaCO_3_ formed, and hence the strength, and the porosity of the geo-material. Consequently, while bacterial calcite precipitation is responsible for improving the mechanical properties of the granular material, control of the hydrogel content and distribution could lead to enticing anti-seepage characteristics.^87^ This example demonstrates the power and versatility of the presented platform technology to enable the fabrication of load-bearing living building materials with thus far inaccessible dynamic functionalities.

## 3 Conclusions

We have introduced a biomimetic regulating technique to control CaCO_3_ through bacteria-laden hydrogel beads, allowing for full control over the location and concentration of bacteria contained in these hydrogels. To achieve this level of control, we used soil-dwelling bacterium *Sporosarcina pasteurii*, which is capable of hydrolysing urea to produce carbonate ions in alkaline conditions. Taking advantage of the calcium binding affinity of certain amino acids present in yeast extract, we introduced a peptide-responsive release mechanism that makes the calcium ions that are weakly bound to the hydrogel matrix readily available for CaCO_3_ precipitation. This concept highlights, for the first time, the potential to program the release of the biochemical machinery of MICP, with the recognition of a component that is inherently specific to the solution used to promote bacterial growth. The potential of this capsule-based MICP technique was showcased *in vitro* and in soil specimens, where hydrogel beads were allowed to degrade upon contact with the YU medium (pH 10) to form CaCO_3_ minerals. Importantly, we demonstrate that alginate encapsulation of *S. pasteurii* allows control over CaCO_3_ phase, morphology and yield. Our concept, integrating MICP with soft materials for controlled dynamic metabolic response and CaCO_3_ structure, constitutes a step change in the design of functional living building materials that can sense, respond and heal without external intervention.

## 4 Experimental section

### 4.1 Materials

*Sporosarcina pasteurii* (strain designation ATCC 11859, CCOS, 2.9 × 10^11^ cfu g^−1^), calcium chloride (Sigma-Aldrich, C4901), yeast extract (Panreac, A1552), ammonium sulphate (Sigma-Aldrich, A4418), trizma base (Sigma-aldrich, 93350), urea (Sigma-Aldrich, 51456), alginic acid sodium salt (low viscosity, Sigma-Aldrich, A1112), carboxymethyl cellulose (250 kDa, Sigma-Aldrich, 419303), ethylenediaminetetraacetic acid (Sigma-Aldrich, E9884).

### 4.2 Preparation of hydrogel beads with immobilised *Sporosarcina pasteurii*

Alginate (3.6% w/v) and carboxymethyl cellulose (3.0% w/w) were investigated as supporting hydrogels. Solutions were sterile-filtered (Sarstedt Filtropur 0.22 μm) before adding bacteria. Freeze–dried *S. pasteurii* (1.5% w/v) was directly incorporated at into the filtered hydrogel solution and homogenised using a magnetic stirrer. Solutions were prepared freshly for use on the same day. A calcium-mediated gelation was used for both hydrogels, prepared by dissolving calcium chloride (1.0 M) in Milli-Q water. This solution was sterilised with a bottle-top vacuum filter (TPP Filtermax, 0.22 μm) before use. The extrusion setup consisted of an infusion pump (Harvard Apparatus PHD Ultra) loaded with a syringe (BD Microlance) containing the polymer-bacteria solution. The syringe was connected to a vertically clamped stainless steel needle (BD Microlance, 25 G) through a polytetrafluoroethylene (PTFE) pipe (ID 14.34 mm) and Luer-Lock PTFE connectors. The distance between the needle tip and the surface of the hardening solution was 5 cm. The hardening bath was magnetically stirred during the extrusion process. The extrusion setup and the extrusion process were placed and conducted under a laminar flow hood and all hardware components were autoclaved at 121 °C for 20 min. Alginate beads were hardened for 30 min, collected through vacuum filtration (TPP Filtermax, 0.22 μm), rinsed with Milli-Q water and stored in Milli-Q water at 4 °C until further use. CMC beads were hardened for 7 days and stored in a CaCl_2_ solution (1 M) at 4 °C until further use.

### 4.3 Rheology

Rheology was performed on disk-shaped hydrogels using a DHR-3 TA instrument with an 8 mm diameter parallel plate steel geometry. All measurements were performed at 25 °C, with an 800 μm gap. Strain–sweep data were obtained using shear mode at a frequency of 10 rad s^−1^.

### 4.4 Viscosity

The viscosity of the alginate and CMC pre-gelled solutions was measured by rheology using a DHR-3 TA instrument equipped with a concentric cylinder. Measurements were conducted at 5% strain and from 0.1 to 100 rad s^−1^. The viscosity value was taken at 10 rad s^−1^. The viscosity of the alginate solution was 22 cP, while that of CMC was 900 cP.

### 4.5 Mechanical characterisation

Uniaxial compression measurements of the hydrogels were performed on a rheometer equipped with a parallel plate geometry (DHR-3, 50 N load cell, TA instrument). Cylindrical hydrogels (*r* = 4 mm, *h* = 2 mm) were compressed at a constant velocity of 1.2 mm min^−1^ until 60% strain was reached. The compressive modulus was calculated as the slope of the initial linear region ranging between 5–15% strain.

Uniaxial compression measurements of the sand scaffolds were performed on a commercial machine (zwickiLine 5 kN, 5 kN load cell, Zwick Roell). Moulded rectangular prismatic samples (*l*_1_ = 30 mm, *l*_2_ = 30 mm, *h* = 15 mm) were compressed at a constant velocity of 10 mm min^−1^. The tangent compressive modulus was calculated at 50% of the uniaxial compressive strength (UCS).

### 4.6 Determination of the calcium content

The calcium content of the Ca–aginate and Ca–CMC beads was determined by inductively coupled plasma optical emission spectrometer (Shimadzu ICPE-9000). Beads (1 g) were dissolved in 10 mL of calcium ion chelator, prepared by dissolving EDTA (0.05 M) in Milli-Q water. The solution containing the free calcium ions crosslinked within the hydrogel was analysed. All measurements were performed in triplicates. The CaCl_2_-eq. molarity was subsequently calculated based on the calcium content of the beads produced, listed in Table S3.†

### 4.7 Preparation of yeast extract-calcium chelate

The yeast extract–calcium complex was prepared by mixing yeast extract (20 g L^−1^), trizma base (0.13 M), and CaCl_2_ (200 mM). After 60 minutes, the mixture was centrifuged (4500 g, 2 min, 23 °C, Eppendorf 5810R) and the supernatant removed to collect the precipitate. The precipitate was resuspended in Milli-Q water and dried in the oven at 50 °C for 12 h before analysis. FTIR on the calcium–yeast complex was conducted on a Nicolet 6700 Thermo Fisher Scientific in the range 400–4000 cm^−1^, 64 scans with resolution 4 cm^−1^. FTIR spectrum of yeast as-received was acquired for comparison.

### 4.8 Live staining fluorescence imaging of *S. pasteurii* in alginate-based hydrogels

*S. pasteurii* was incubated in NH_4_–YE medium (ATCC 1376) for 24 h at 30 °C. Following incubation, 250 μL of solution was resuspended in 250 μL of fresh NH_4_–YE medium and 1 μL of BactoView Live Green (40102, Biotium) was added to the solution. This solution was subsequently incubated at room temperature and protected from light for an additional 30 min. To remove unreacted dye, the sample was centrifuged for 3 min at 12 000 rcf, the supernatant removed, and the pellet resuspended in 250 μL of fresh NH_4_–YE medium. This process was repeated 3 times.

To encapsulate stained bacteria in the hydrogel, 100 μL of stained sample was mixed with 1 mL of 3.6% w/v sodium alginate solution. The solution was gelled for 30 min in a 1 M CaCl_2_ bath. Live staining fluorescence imaging was performed using a Nikon Eclipse Ni microscope.

### 4.9 Assessment of bacterial cell growth and viability

The survival and metabolic activity of bacteria after the extrusion process was assessed by dissolving bacteria-laden hydrogel beads (0.5 g) in 50 mL of YU medium (20 g L^−1^ urea, 3 g L^−1^ yeast extract, and 0.13 M trizma base) and incubated at 30 °C for 24 h. After incubation, bacterial growth and viability were determined by re-suspending in 30 mL of NH_4_–YE medium (ATCC 1376) to an optical density measured at a wavelength of 600 nm (OD_600_) of 0.01. Samples were subsequently incubated at 30 °C, and OD_600_ and urease activity values monitored over a one week period. The encapsulation yield (EY) was calculated as the ratio of the OD_600_ of the microencapsulated cells to that of non-immobilised freeze–dry cells (OD_600,FD_) after 2 days:3
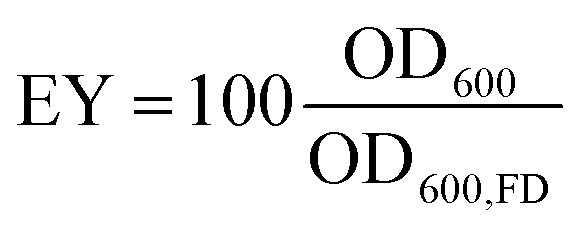


Urease activities of bacterial cells are thus determined by measuring the relative change in conductivity when the bacterial solution is exposed to urea (1.11 M) for a 5 min-duration.^88^ Subsequently, the rate of electrical conductivity increase (mS min^−1^) is converted to urea hydrolysis rate (mM h^−1^) using eqn (5).

4 Urea hydrolysed (mM) = conductivity (mS)·11.11

5
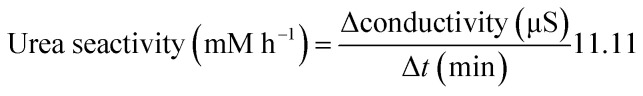


Bacterial abundance following release from the hydrogel into the YU medium was determined using flow cytometry (NovoCyte, ACEA Biosciences) on cells fixed formaldehyde (3.7% final concentration) and stained with SYTO13 solution (2.5 μM, ThermoFisher) for 15 min.

### 4.10 Precipitation experiments

Calcium carbonate (CaCO_3_) precipitation experiments were performed at ambient temperature in 14 mL Falcon tubes. Solutions of YU medium, composed of yeast extract (3 g L^−1^), increasing urea concentrations (0.01, 0.1, 0.3, 1 M) and trizma base (0.13 M) were sterile-filtered (TPP Filtermax, 0.22 μm) and poured into each tube to a volume of 10 mL. Beads containing 0.0005–0.2 M CaCl_2_-eq. were subsequently introduced into each tube in the YU solution, and preserved in the mother culture medium at room temperature for 7 days. For example, because 1 M CaCl_2_ contains 40 g L^−1^ of calcium, 1 g of beads containing 0.40 ± 0.03 g L^−1^ corresponds to a 0.01 M CaCl_2_-eq. molarity. The heat maps in Fig. S6† schematically show the ratio of urea/CaCl_2_-eq. molarity for both hydrogels. The adopted notation refers to the supporting polymer, and the CaCl_2_-eq. and urea molarities. For example, Alg-0.002 M-0.01 M refers to alginate beads dissolved in a 0.002 M CaCl_2_-eq. and 0.01 M urea solution.

To collect the precipitated solids, the solution was centrifuged (4500 g, 2 min, 23 °C; Eppendorf 5810R) and, following the removal of the supernatant, rinsed with distilled water and vortexed for 2 min. This procedure was performed twice to form a CaCO_3_ suspension solution, which was decanted for drying at 50 °C for 48 h. We assume that the removal of the CaCO_3_ particles from the crystallising solution stops the growth and/or phase transformation of the CaCO_3_ minerals as no additional urea is supplied any more.^66,89^*Ex situ* characterisation of the evolution of solid products collected from filtration was performed with thermogravimetric analysis (TGA), X-ray powder diffraction (XRD), FTIR, and scanning electron microscopy with energy-dispersive X-ray spectroscopy (SEM/EDX).

### 4.11 Preparation of soil specimens

Medium grained sand with properties shown in Table S9† was used. The sand was supplied uncrushed, dry and washed by Carlo Bernasconi SA (Switzerland). Thus, it was free of organics, clay or silt. Beads (10 wt% of dry sand) were mixed with the soil, and the mixture packed in a silicone mould to the target dry density by gently tapping their side with a tamping rod. Specimens were submerged for 48 h in a YU medium bath (3 g L^−1^ yeast extract, 1 M urea, 0.13 M trizma base) to dissolve the beads and cured for an additional 10 days with a total of 100 mL of a cementation solution containing an equimolar amount of urea and CaCl_2_ (1 M), and yeast extract (3 g L^−1^), all dissolved in MilliQ water. Specimens were dried at 100 °C for 24 h. *Ex situ* characterisation of the three-dimensional (3D) pore space and the volume of CaCO_3_ formed was performed with X-ray μ-CT (XRCT) and thermogravimetric analysis (TGA).

### 4.12 Characterisation of precipitated solids

#### 4.12.1 Thermogravimetric analysis (TGA)

Experiments were performed using a TGA 4000 Thermogravimetric Analyser from PerkinElmer. Around 2–10 mg of sample were placed in a cylindrical 146 μL ceramic crucible (ID 7.18 mm, depth 4.79 mm). The TGA furnace was constantly purged with 20 mL min^−1^ of N_2_ gas. Samples were heated from 30 °C to 950 °C at a heating rate of 10  °C min^−1^ in a stream of N_2_ “reactive gas” provided directly above the sample with a flow rate of 20 mL min^−1^. A baseline, obtained under the same conditions with empty ceramic crucibles, was subtracted from the measured thermograms.

#### 4.12.2 X-Ray powder diffraction (XRD)

Measurements were performed using an Empyrean, Malvern Panalytical equipped with a copper sealed tube X-ray source producing CuKα radiation at a wavelength of 1.5406 Å from a generator operating at 40 keV and 40 mA. Scanning rate was 0.03° 2*Θ* per minute from 15 to 50°.

#### 4.12.3 Fourier-transform infrared spectroscopy (FTIR)

IR analysis were performed using a Nicolet 6700 spectrometer from Thermo Fisher Scientific. For each sample 64 scans were recorded in the 400–4000 cm^−1^ spectral range with a resolution of 4 cm^−1^.

#### 4.12.4 Scanning electron microscopy (SEM)

Scanning electron microscopy (SEM) was performed on a Zeiss Gemini 300 at 1 kV using secondary electrons detector. Samples were coated with 5 nm of gold.

#### 4.12.5 Energy-dispersive X-ray spectroscopy (EDX)

Energy-dispersive X-ray spectroscopy (EDX) was conducted on a Zeiss Gemini 300 equipped with an Oxford Instrument EDX detector. Accelerating voltage was 14 kV, probe current was 4 nA. Samples were coated with 10 nm of carbon.

#### 4.12.6 Optical microscopy

Optical microscopy imaging was performed with an automated epifluorescence microscope (AxioImager.Z2, Zeiss) equipped with a digital camera (AxioCam 305 mono, Zeiss) after staining of cellular DNA with 1X SybrGreen (ThermoFisher) for 15 min at room temperature.

#### 4.12.7 X-Ray μ-CT (XRCT)

X-Ray μ-CT (XRCT performed with an Ultratom μ-tomography system (RX-SOLUTIONS). The volume, with a physical size of 5.88 mm (*W*) × 5.53 mm (*D*) × 5.28 mm (*H*), was scanned at a voxel resolution of 5.01 μm, with an energy of 55 kV and a current of 70 μA. Amira-Avizo v. 2019.4 was used for reconstruction, segmentation, particle analysis and visualisation.

## Author contributions

A. C. S. and L. L. conceptualised the experimental program. A. C. S. and L. L. immobilised and cultivated the bacteria and performed *in vitro* precipitation. L. L. and M. H. performed the calcium-binding yeast extract experiments and helped A. C. S. with data interpretation and visualisation. L. L. conducted the characterisation of CaCO_3_ minerals and helped A. C. S. with data interpretation and visualisation. L. L. performed the staining fluorescent and time-lapse imaging of hydrogels. H. M. P. performed the optical microscopy imaging and collected flow cytometry data of released bacteria, and helped A. C. S. with data interpretation. A. C. S. and L. L. prepared the soil-based specimens. A. C. S. and D. T. performed the XRCT analyses and interpretation. A. C. S., L. L. and M. H. performed the mechanical and rheological characterisations. Lyesse Laloui and D. T. acquired the financial support for the project. A. C. S administered the project, performed the data curation and visualisation, and wrote the manuscript with the help of L. L., M. H. and E. A. All authors reviewed the manuscript.

## Conflicts of interest

There are no conflicts to declare.

## Supplementary Material

TA-009-D1TA03990C-s001
