# Supplementary material for: Controlling the calcium carbonate microstructure of engineered living building materials
Source: J Mater Chem A Mater. 2021 Oct 29;9(43):24438–51. doi: 10.1039/d1ta03990c (PMC8577622; doi:10.1039/d1ta03990c)
Supplement: TA-009-D1TA03990C-s001 [file TA-009-D1TA03990C-s001.pdf]

Electronic Supplementary Material (ESI) for Journal of Materials Chemistry A.
This journal is © The Royal Society of Chemistry 2021

Supplementary Information

Controlling the calcium carbonate microstructure of engineered living building materials

Alexandra Clarà Saracho,*[a] Lorenzo Lucherini,[a] Matteo Hirsch,[b] Hannes M. Peter,[c] Dimitrios Terzis,[a] Esther Amstad,[b] and Lyesse Laloui[a]

Table S1. Extrusion dripping process parameters.

Process parameter	Value
Flow rate	$1000\,\mu\mathrm{L\,min^{-1}}$
Drop height	5 cm
Stirring rate of gelation bath	200 rpm
Hardening time Alginate	30 min
Hardening time CMC	7 d

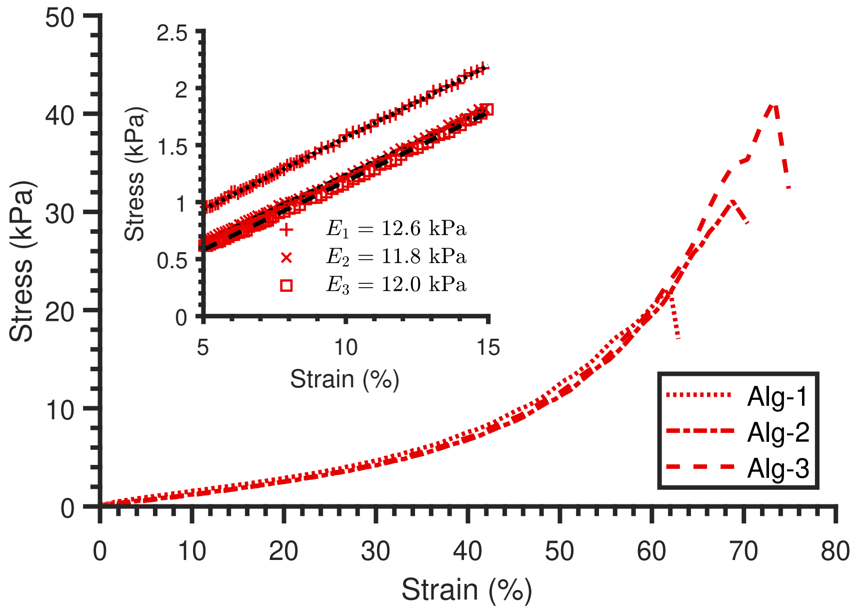

Fig. S1. Stress-strain curves for alginate under uniaxial compression ($n = 3$).

Table S2. Stress-strain parameters for alginate under uniaxial compression ($n = 3$) with calculated mean (μ) and standard deviation (σ^2).

Sample ID	Peak stress, σ_p (kPa)	Peak strain, ϵ_p (%)	Compressive modulus, E (kPa)
Alg-1	22.0	62.0	12.6 ($R^2 = 0.9994$)
Alg-2	31.1	68.8	11.8 ($R^2 = 0.9991$)
Alg-3	41.5	73.4	12.0 ($R^2 = 0.9987$)
μ	31.5	68.0	12.1
σ^2	9.7	4.7	0.4

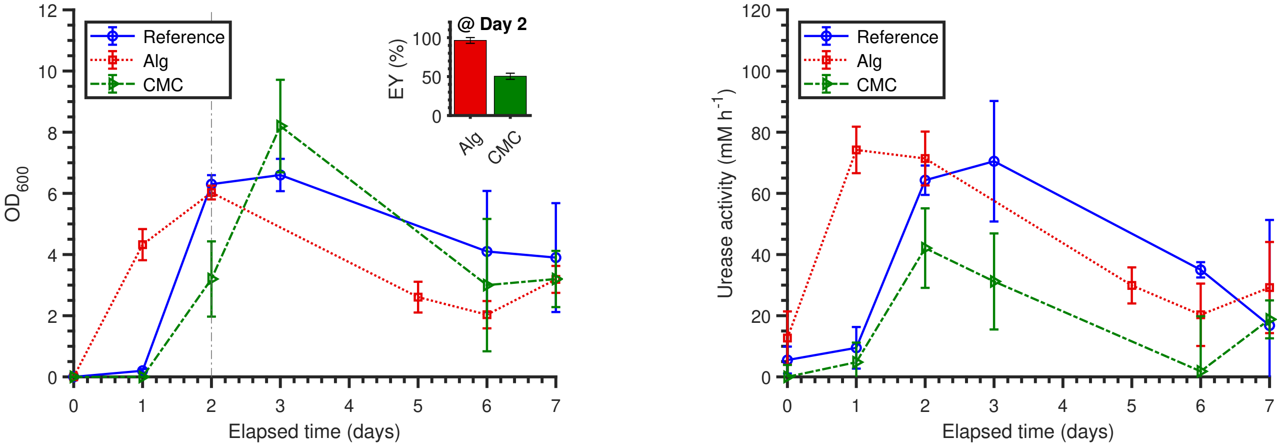

(A)

(B)

Fig. S2. Viability of *S. pasteurii* after the extrusion process: (A) growth curves and encapsulation yield (EY, zoomed in plot), and (B) urease activity of immobilised and mobile (Reference) bacteria.

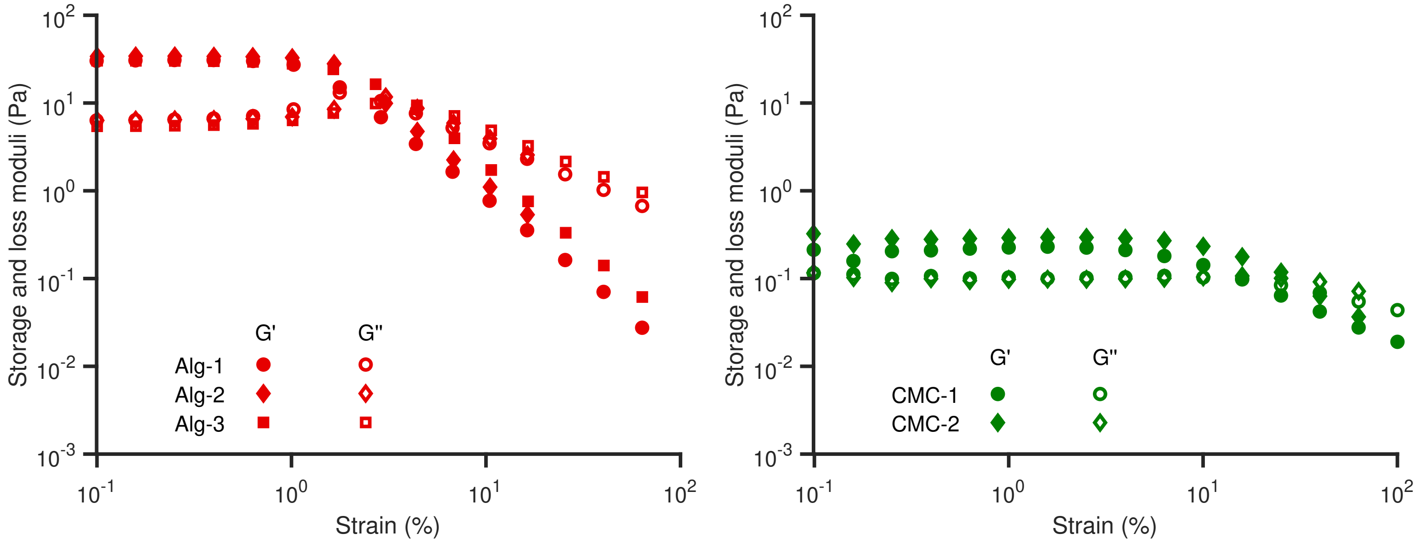

(A)

(B)

Fig. S3. Rheological analyses of (A) alginate ($n = 3$) and (B) CMC ($n = 2$) hydrogels.

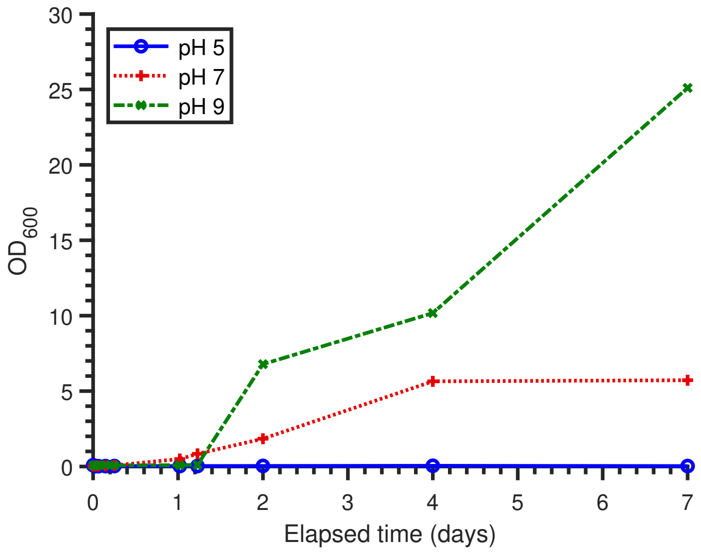

Fig. S4. pH effect on the growth curve of *S. pasteurii* cultivated in NH_4—YE medium.

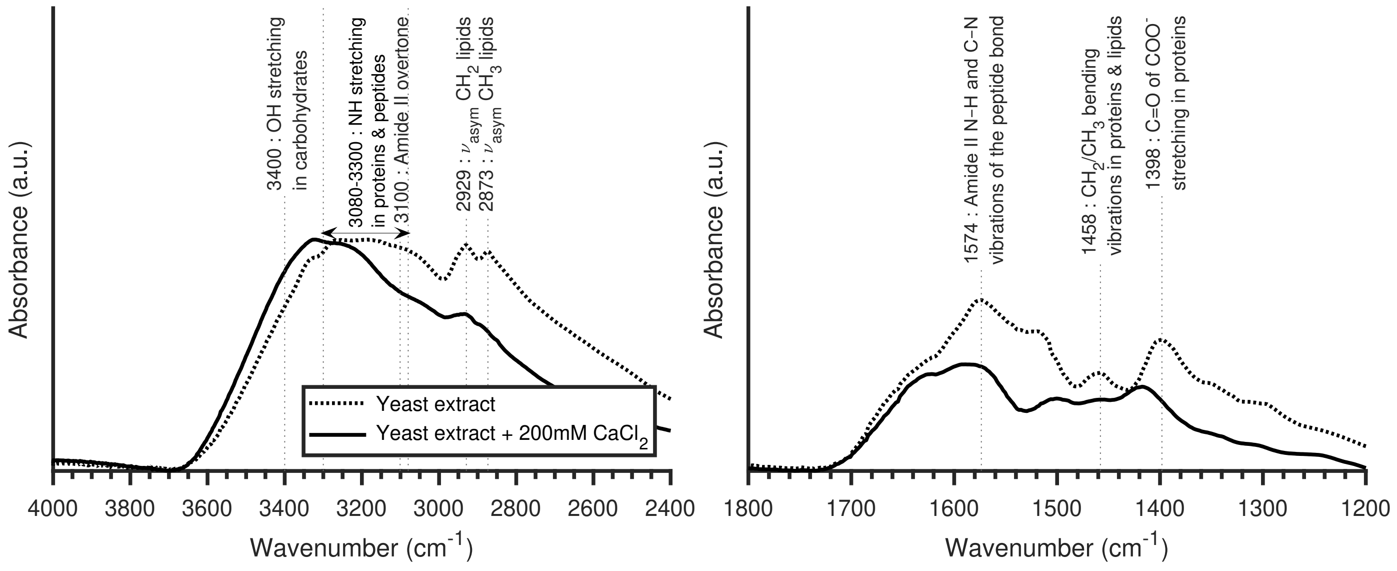

(A)

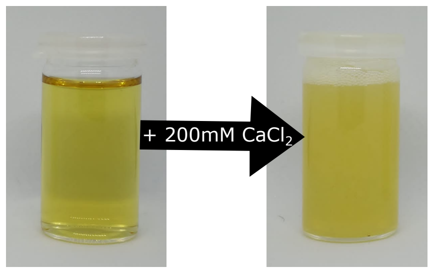

(B)

Fig. S5. (A) FTIR spectra of yeast extract and yeast extract-calcium chelate in the spectral windows $4000-2400\,cm^{-1}$ (left) and $1800-1200\,cm^{-1}$ (right). (B) Photographs showing the increase in the turbidity of the solution after formation of the peptide-calcium chelate.

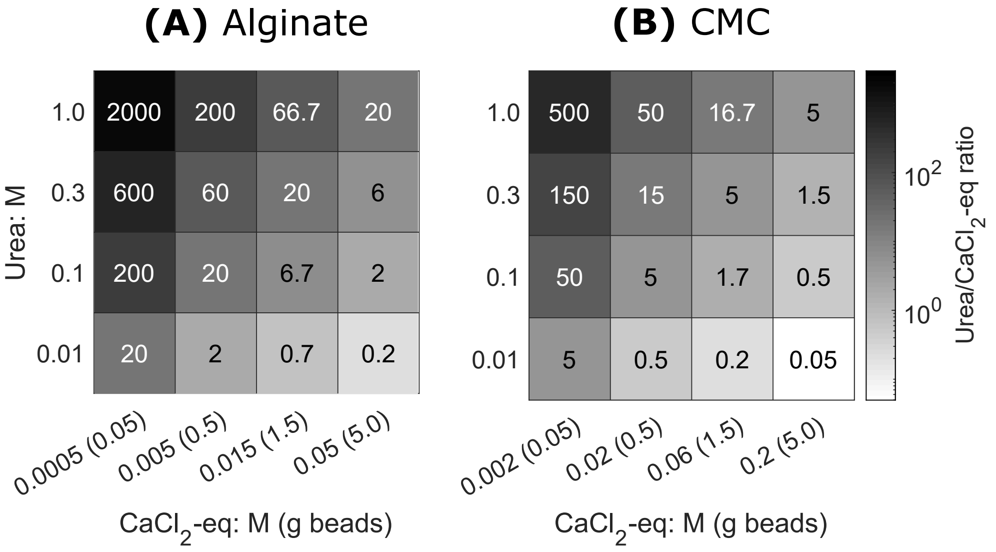

Fig. S6. Heat map of the sample identification notation adopted corresponding to the ratio of urea/$CaCl_2$—eq molarity for alginate and CMC hydrogels. Note that the urea/$CaCl_2$—eq ratio remains constant along the diagonal. The adopted sample identification notation refers to the hydrogel, and the $CaCl_2$—eq and urea concentrations used. For example, Alg-$0.05\,\text{M}$-$1.0\,\text{M}$ refers to bacteria immobilised in alginate beads, and released in a $0.05\,\text{M}$ $CaCl_2$—eq and $1.0\,\text{M}$ urea solution.

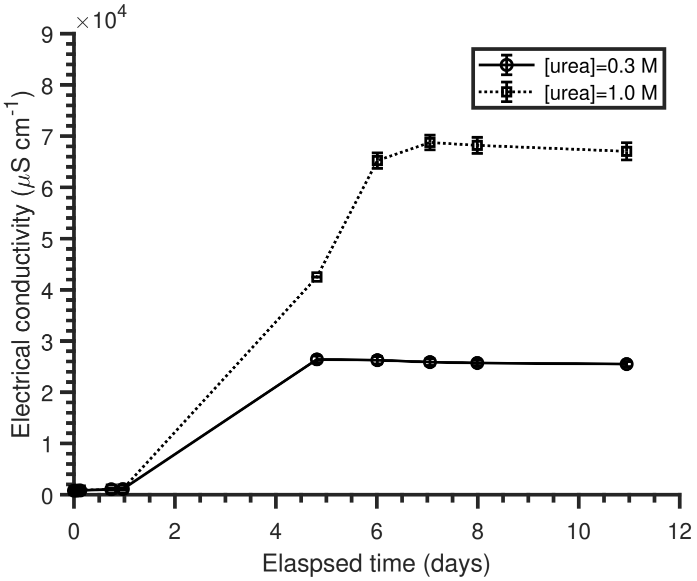

Fig. S7. Electrical conductivity over time resulting from the hydrolysis of urea by *S. pasteurii* released from the alginate beads in two solutions with different initial urea concentrations (0.3 and $1.0\,\text{M}$). Higher electrical conductivity values resulting from the larger amount of CO_3^{2-} from urea hydrolysis are observed at higher urea concentration.

Table S3. Calcium content of hydrogel beads (values per 1 g of beads, $n = 3$).

Supporting polymer	Alg	CMC
Ca concentration ($\mathrm{g\,L^{-1}}$)	0.40 ± 0.03	1.56 ± 0.61
$CaCl_2$−eq (M)	0.01	0.04

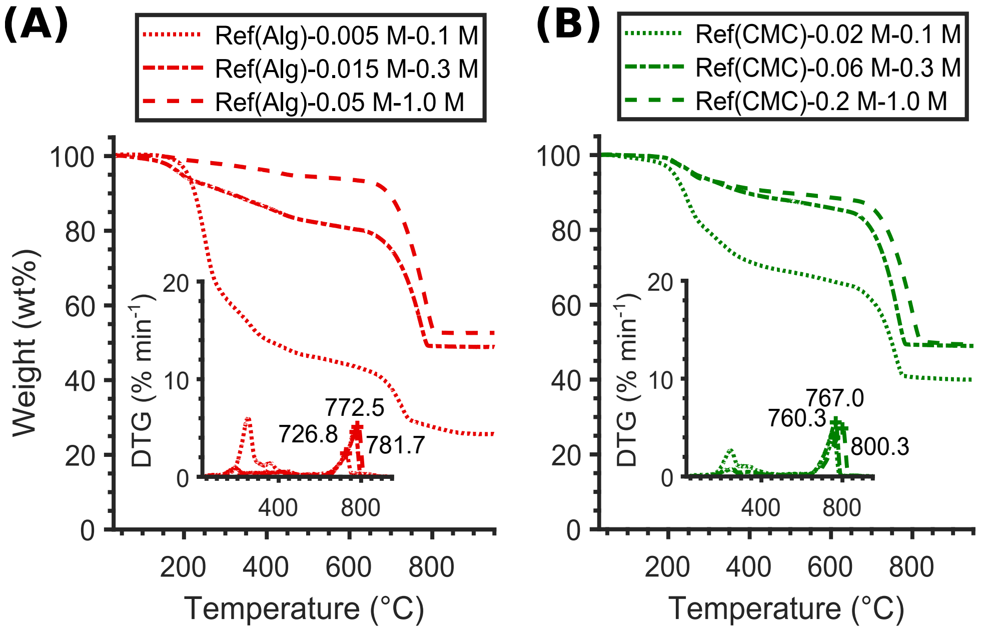

Fig. S8. Thermogravimetric analyses with peak $CaCO_3$ decomposition temperature indicated showing the compositional changes of $CaCO_3$ formed in the absence of hydrogels (Reference): (A) alginate-free and (B) CMC-free.

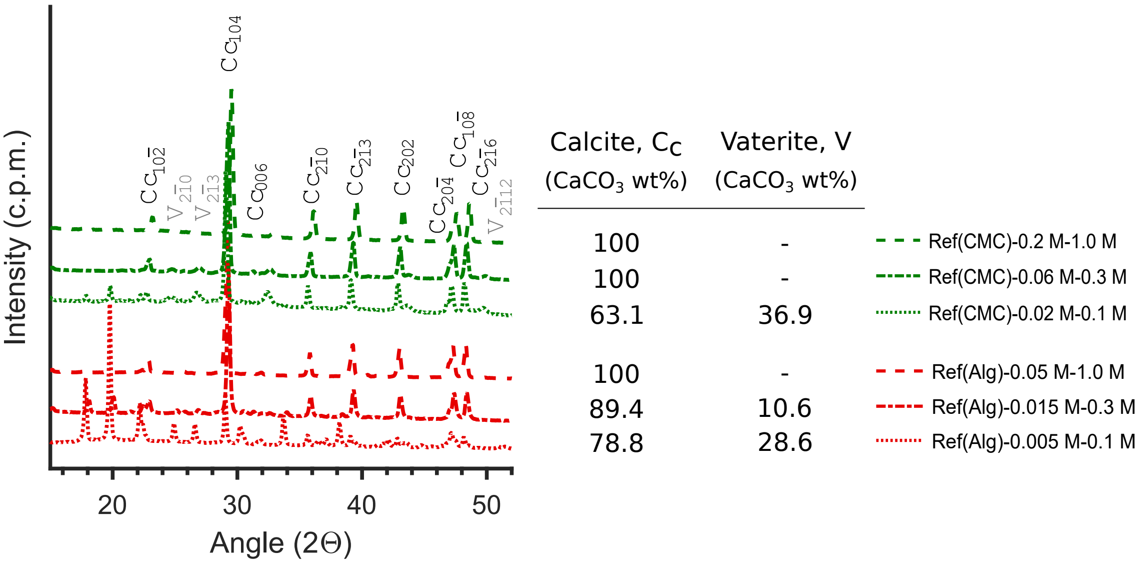

Fig. S9. XRD patterns of $CaCO_3$ formed in the absence of hydrogels (Reference) with hkl values of Bragg peaks and relative mineral phase abundance indicated.

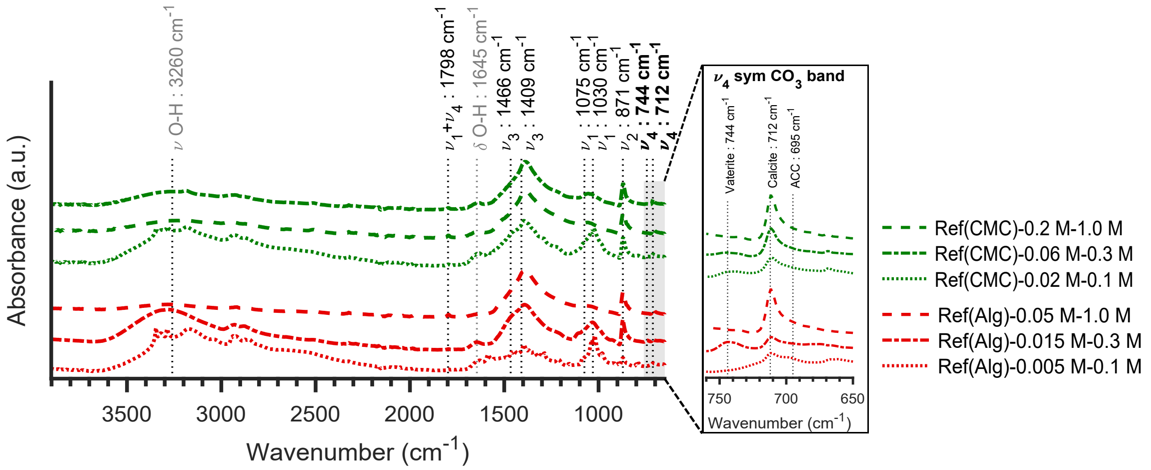

Fig. S10. Infrared spectra showing the structural changes of $CaCO_3$ formed in the absence of hydrogels (Reference).

Table S4. Summary of the FTIR peak intensities of $CaCO_3$ formed in the absence of hydrogels.

Sample ID	$\nu_3/\nu_{2,H_2O}$	ν_3/ν_1	ν_3/ν_2	ν_3/ν_4
Ref(Alg)-0.005 M-0.1 M	2.01	1.65	1.67	2.29
Ref(Alg)-0.015 M-0.3 M	4.19	2.34	1.46	3.42
Ref(Alg)-0.05 M-1.0 M	9.46	3.61	1.76	3.78
Ref(CMC)-0.02 M-0.1 M	2.99	1.72	1.54	3.09
Ref(CMC)-0.06 M-0.3 M	6.22	2.86	1.78	4.35
Ref(CMC)-0.2 M-1.0 M	5.36	2.74	1.74	3.38

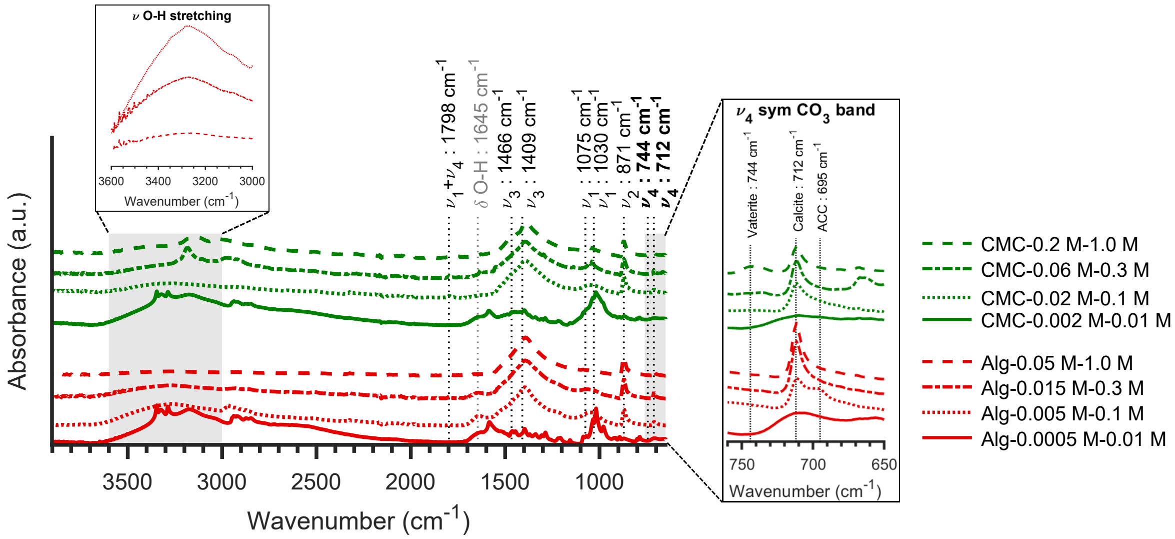

Fig. S11. Infrared spectra showing the structural changes of $CaCO_3$ formed in the presence of hydrogels.

Table S5. Summary of the FTIR peak intensities of $CaCO_3$ formed in the presence of hydrogels.

Sample ID	$\nu_3/\nu_{2,H_2O}$	ν_3/ν_1	ν_3/ν_2	ν_3/ν_4
Alg-0.0005 M-0.01 M		n/a		
Alg-0.005 M-0.1 M	3.91	2.92	2.31	10.10
Alg-0.015 M-0.3 M	5.69	4.04	1.91	5.83
Alg-0.05 M-1.0 M	21.39	7.25	2.00	5.55
CMC-0.002 M-0.01 M	1.52	n/a	1.44	1.67
CMC-0.02 M-0.1 M	10.80	2.71	1.72	3.89
CMC-0.06 M-0.3 M	n/a	n/a	2.13	4.13

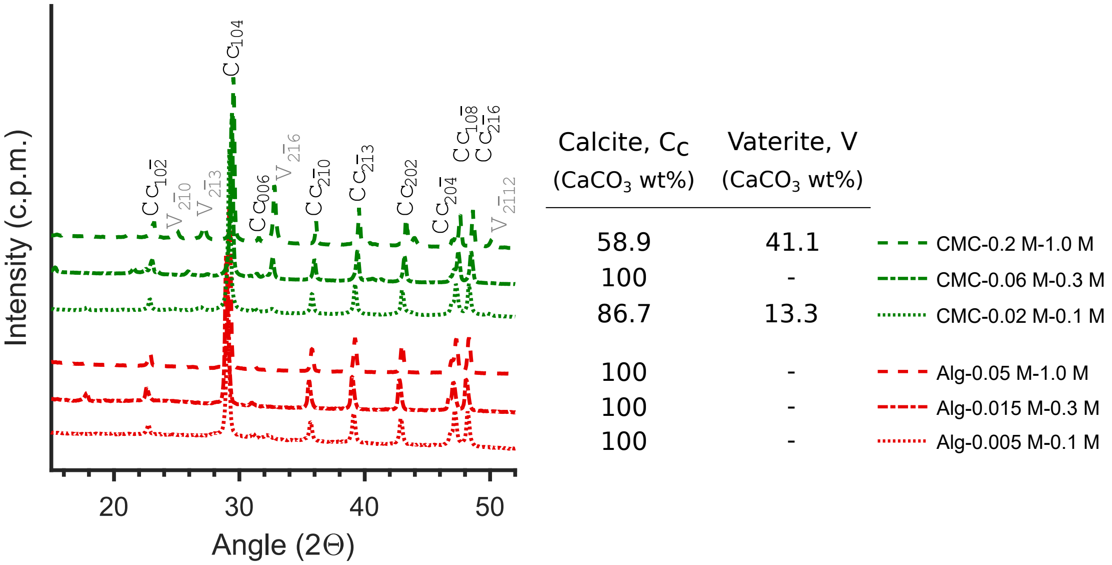

Fig. S12. XRD patterns of $CaCO_3$ formed in the presence of hydrogels with hkl values of Bragg peaks and relative mineral phase abundance indicated.

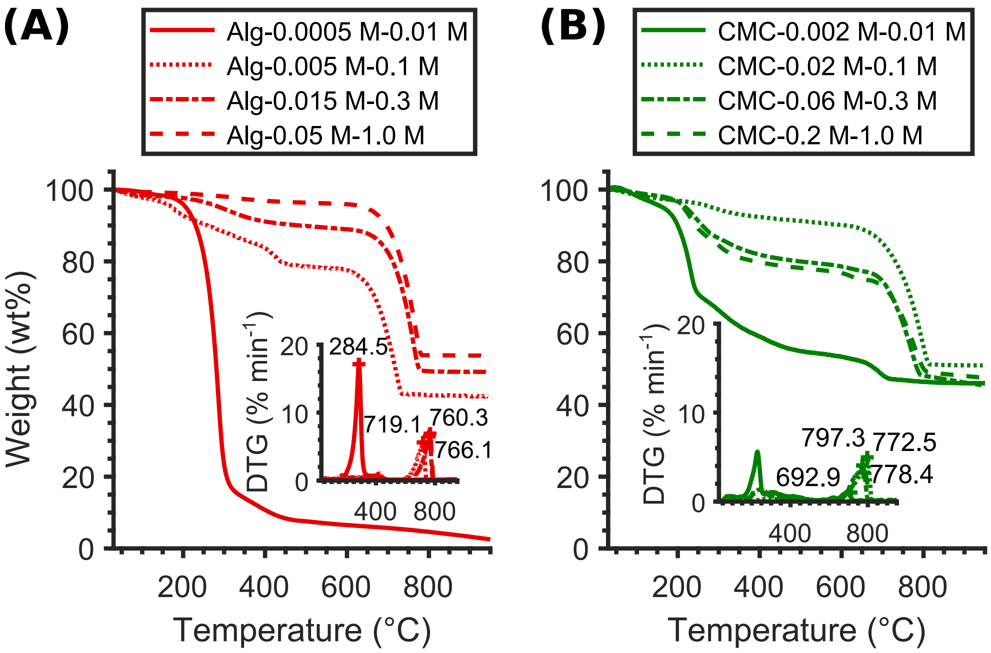

Fig. S13. Thermogravimetric analyses with peak $CaCO_3$ decomposition temperature indicated showing the compositional changes of $CaCO_3$ formed in the presence of hydrogels: (A) alginate and (B) CMC.

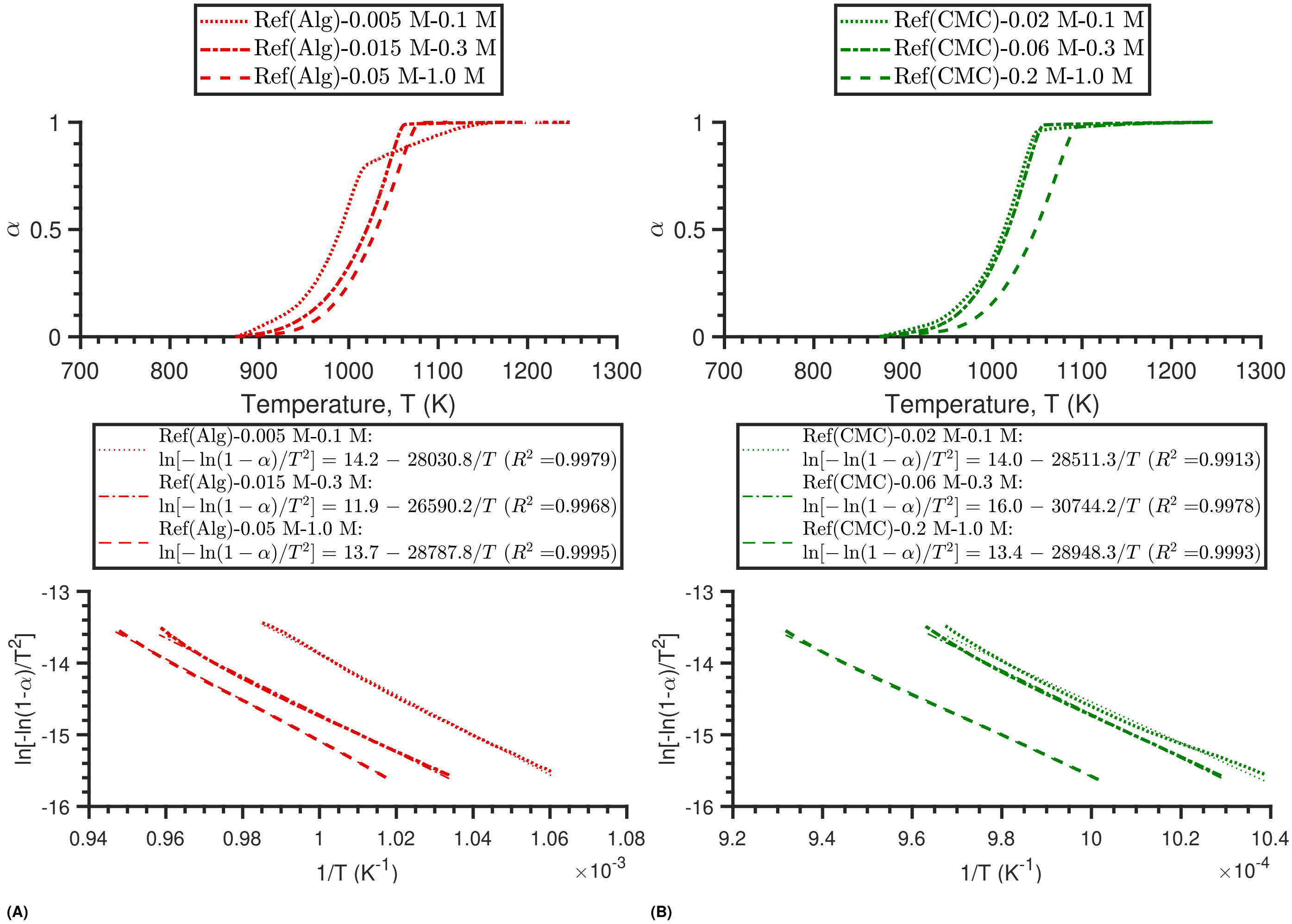

Fig. S14. (Top) α vs. temperature plots and (Bottom) Coats-Redfern kinetics model of the decomposition of $CaCO_3$ precipitated in the absence of hydrogel (Reference; $\alpha = 0.15 - 0.78$ [67]): (A) Ref(Alg) and (B) Ref(CMC).

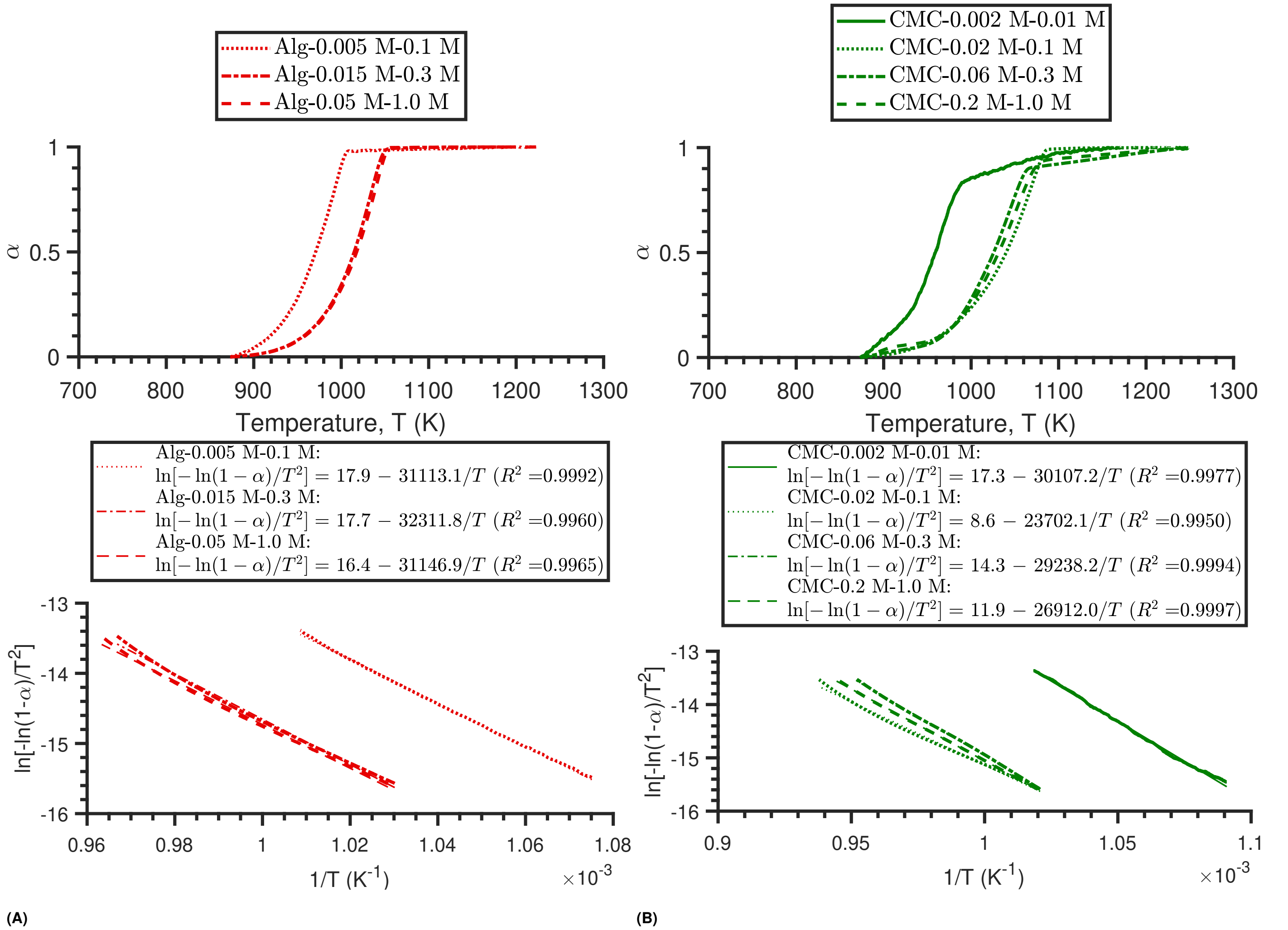

Fig. S15. (Top) α vs. temperature plots and (Bottom) Coats-Redfern kinetics model of the decomposition of $CaCO_3$ precipitated in the presence of hydrogel ($\alpha = 0.15 - 0.78$ [67]): (A) alginate and (B) CMC.

Table S6. TGA and kinetic parameters of the $CaCO_3$ decomposition in the absence of hydrogel (Reference).

Sample ID	Peak temperature, T (°C)	Calcination weight loss ($CaCO_3$ content) (wt%)	Activation energy, E_a (kJ mol^{-1})	Pre-exponential factor, ln A (min^{-1})	R^2
Ref(Alg)-0.005 M-0.1 M	749.5 ± 32.4	24.9 ± 10.1 (64.3 ± 24.1)	222.1 ± 10.6	28.2 ± 1.9	0.9990 ± 0.0004
Ref(Alg)-0.015 M-0.3 M	738.9 ± 2.5	26.4 ± 2.4 (63.7 ± 8.7)	254.2 ± 18.4	32.3 ± 2.7	0.9984 ± 0.0021
Ref(Alg)-0.05 M-1.0 M	782.5 ± 1.1	38.6 ± 3.7 (87.3 ± 9.8)	249.4 ± 14.3	30.8 ± 1.7	0.9999 ± 0.0000
Ref(CMC)-0.02 M-0.1 M	760.1	26.6 (67.8)	250.6	31.5	0.9934
Ref(CMC)-0.06 M-0.3 M	776.3 ± 13.3	26.7 ± 2.5 (74.2 ± 0.7)	238.9 ± 23.6	29.5 ± 3.5	0.9987 ± 0.0012
Ref(CMC)-0.2 M-1.0 M	809.5 ± 13.0	32.4 ± 8.7 (82.4 ± 9.0)	235.1 ± 7.9	28.2 ± 1.5	0.9988 ± 0.0007

Table S7. TGA and kinetic parameters of the $CaCO_3$ decomposition in the presence of alginate. Sample IDs in bold refer to the values along the diagonal displayed in Figure S6.

Sample ID	Peak temperature, T (°C)	Calcination weight loss ($CaCO_3$ content) (wt%)	Activation energy, E_a (kJ mol^{-1})	Pre-exponential factor, ln A (min^{-1})	R^2
**Alg-0.0005 M-0.01 M**	n/a	0.0	n/a	n/a	n/a
Alg-0.0005 M-0.1 M		0.0			
Alg-0.0005 M-0.3 M		0.0			
Alg-0.0005 M-1.0 M		0.0			
**Alg-0.005 M-0.1 M**	713.3 ± 12.2	38.7 ± 3.8 (82.0 ± 3.6)	249.6 ± 13.8	33.0 ± 1.9	0.9986 ± 0.0017
Alg-0.005 M-0.3 M	684.3 ± 22.4	29.7 ± 5.0 (70.0 ± 26.1)	307.2 ± 29.8	41.2 ± 4.7	0.9968 ± 0.0018
Alg-0.005 M-1.0 M	719.6 ± 4.7	36.8 ± 3.1 (86.3 ± 3.9)	266.2 ± 3.6	34.7 ± 0.4	0.9975 ± 0.0017
Alg-0.015 M-0.01 M	749.8 ± 7.5	39.4 ± 0.6 (90.7 ± 1.6)	270.2 ± 3.2	34.3 ± 0.6	0.9963 ± 0.0015
**Alg-0.015 M-0.3 M**	761.0 ± 1.0	40.1 ± 0.2 (89.6 ± 0.6)	268.3 ± 8.2	33.7 ± 1.0	0.9967 ± 0.0010
Alg-0.015 M-1.0 M	747.9 ± 8.6	40.0 ± 0.7 (87.5 ± 3.4)	282.6 ± 6.2	35.8 ± 1.0	0.9981 ± 0.0002
Alg-0.05 M-0.3 M	785.3	38.5 (88.0)	267.3	32.8	0.9973
**Alg-0.05 M-1.0 M**	773.5 ± 10.3	42.6 ± 0.6 (96.6 ± 0.6)	254.5 ± 5.0	31.6 ± 1.2	0.9973 ± 0.0014

Table S8. TGA and kinetic parameters of the $CaCO_3$ decomposition in the presence of CMC. Sample IDs in bold refer to the values along the diagonal displayed in Figure S6.

Sample ID	Peak temperature, T (°C)	Calcination weight loss ($CaCO_3$ content) (wt%)	Activation energy, E_a (kJ mol^{-1})	Pre-exponential factor, ln A (min^{-1})	R^2
**CMC-0.002 M-0.01 M**	692.9	5.4 (52.7)	170.4	22.15	0.9963
CMC-0.002 M-0.1 M	746.3 ± 1.9	23.8 ± 0.3 (63.9 ± 0.4)	252.2 ± 28.0	32.0 ± 3.5	0.9980 ± 0.0006
CMC-0.002 M-0.3 M	740.0 ± 7.3	30.3 ± 0.5 (70.5 ± 2.9)	269.0 ± 21.8	34.2 ± 2.4	0.9982 ± 0.0013
CMC-0.002 M-1.0 M	737.3 ± 19.6	25.2 ± 1.6 (60.4 ± 0.2)	234.2 ± 4.5	29.8 ± 0.0	0.9993 ± 0.0004
CMC-0.02 M-0.01 M	750.3	13.5 (52.5)	201.2	25.2	0.9997
**CMC-0.02 M-0.1 M**	791.5	30.6 (82.0)	197.1	24.3	0.9950
CMC-0.02 M − 0.3 M	811.3	41.5 (92.9)	207.8	25.2	0.9962
CMC-0.02 M-1.0 M	774.7	41.3 (93.9)	217.5	27.3	0.9986
CMC-0.06 M-0.01 M	737.3	8.1 (47.6)	139.2	17.5	0.9986
**CMC-0.06 M-0.3 M**	772.3	28.6 (77.4)	243.1	30.2	0.9994
CMC-0.06 M-1.0 M	772.4	22.4 (69.9)	244.2	30.3	0.9997
**CMC-0.2 M-1.0 M**	778.4	25.5 (74.8)	223.7	27.7	0.9997

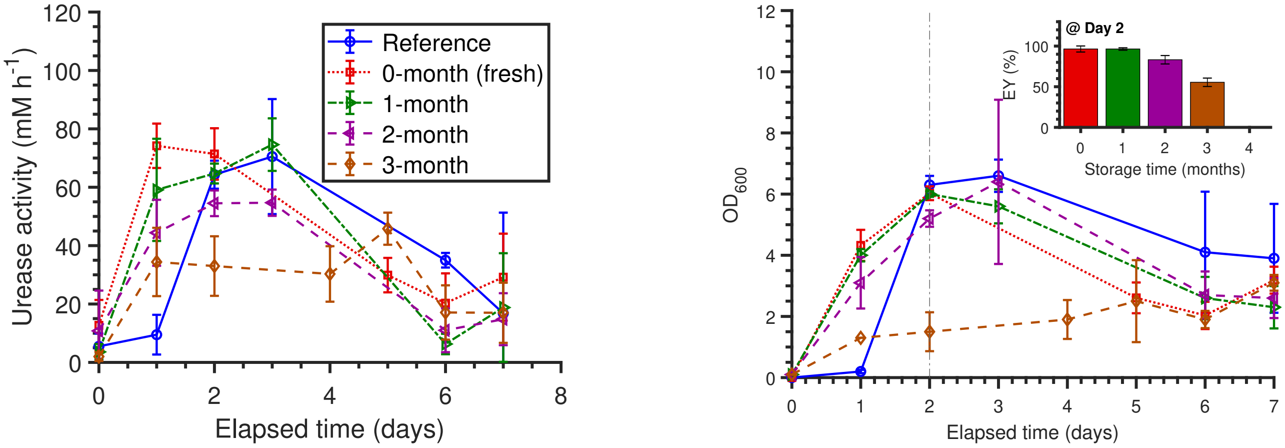

(A) **(B)**

Fig. S16. Shelf-life of *Sporosarcina pasteurii* embedded within alginate beads stored at 4 ℃ for a 4-month period: (B) urease activity, and (A) growth curves and encapsulation yield (EY, zoomed in plot) of immobilised and mobile (Reference) bacteria.

Table S9. Untreated sand scaffold properties.

Specific gravity, G_s	2.65
Mean particle size, D_{50}: μm	363
Maximum void ratio, e_{max}	0.89
Minimum void ratio, e_{min}	0.69
Dry bulk density, ρ_d: g cm^{-3}	1.55
Void ratio, e	0.71
Porosity, n	0.415

Table S10. MICP-treated sand specimen properties calculated from X-Ray μ-CT data.

$CaCO_3$ content: wt%	0.514
Porosity, n	0.27
Back-calculated alginate content: wt%	14.5

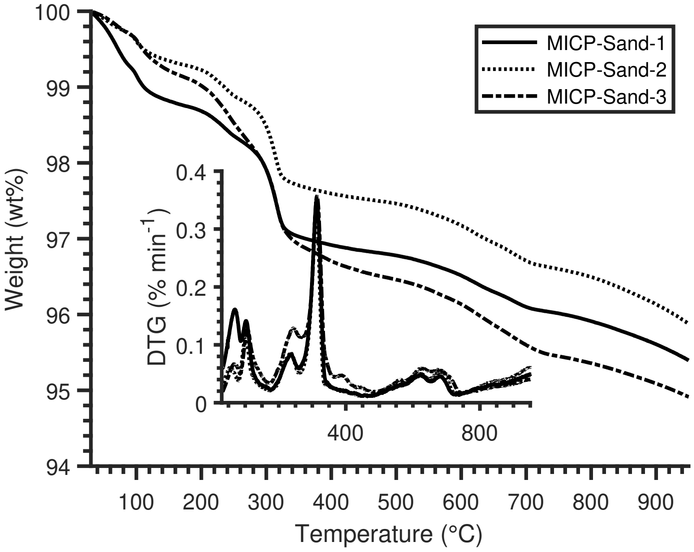

Fig. S17. Thermogravimetric analyses of biomineralised hydrogel-sand scaffolds.

Table S11. TGA parameters of the decomposition of the biomineralised hydrogel-sand scaffolds.

Sample ID	Peak temperature, T (°C)	Calcination weight loss ($CaCO_3$ content) (wt%)
MICP-Sand-1	620.9	0.489 (1.11)
MICP-Sand-2	675.8	0.324 (0.74)
MICP-Sand-3	659.6	0.603 (1.37)
μ	652.1	0.472 (1.07)
σ^2	28.2	0.14 (0.32)

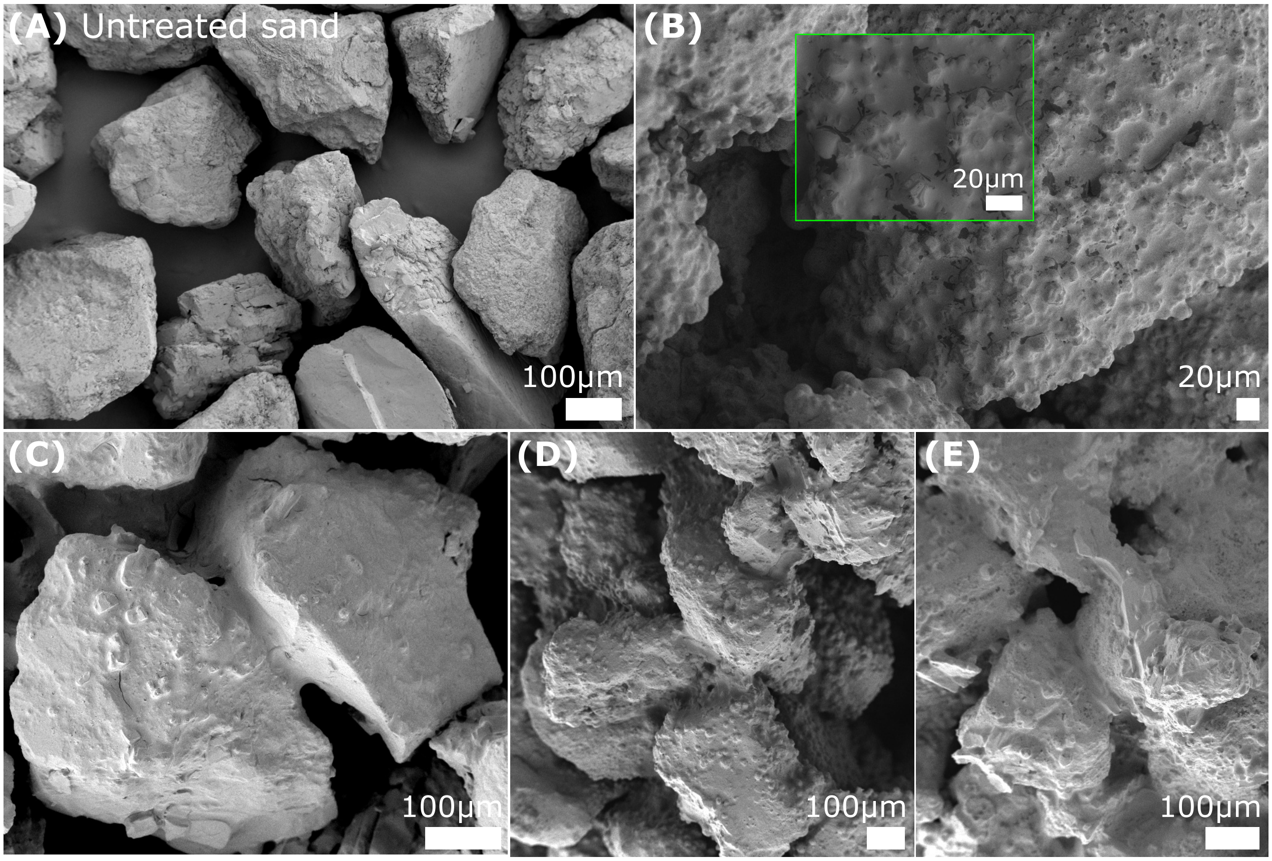

Fig. S18. SEM-SE2 images of the biomineralised hydrogel-sand scaffolds: (A) untreated sand grains; (B) $CaCO_3$ minerals on sand grain surface; and (C-E) alginate bridging sand grains and providing a substrate for $CaCO_3$ mineralisation.

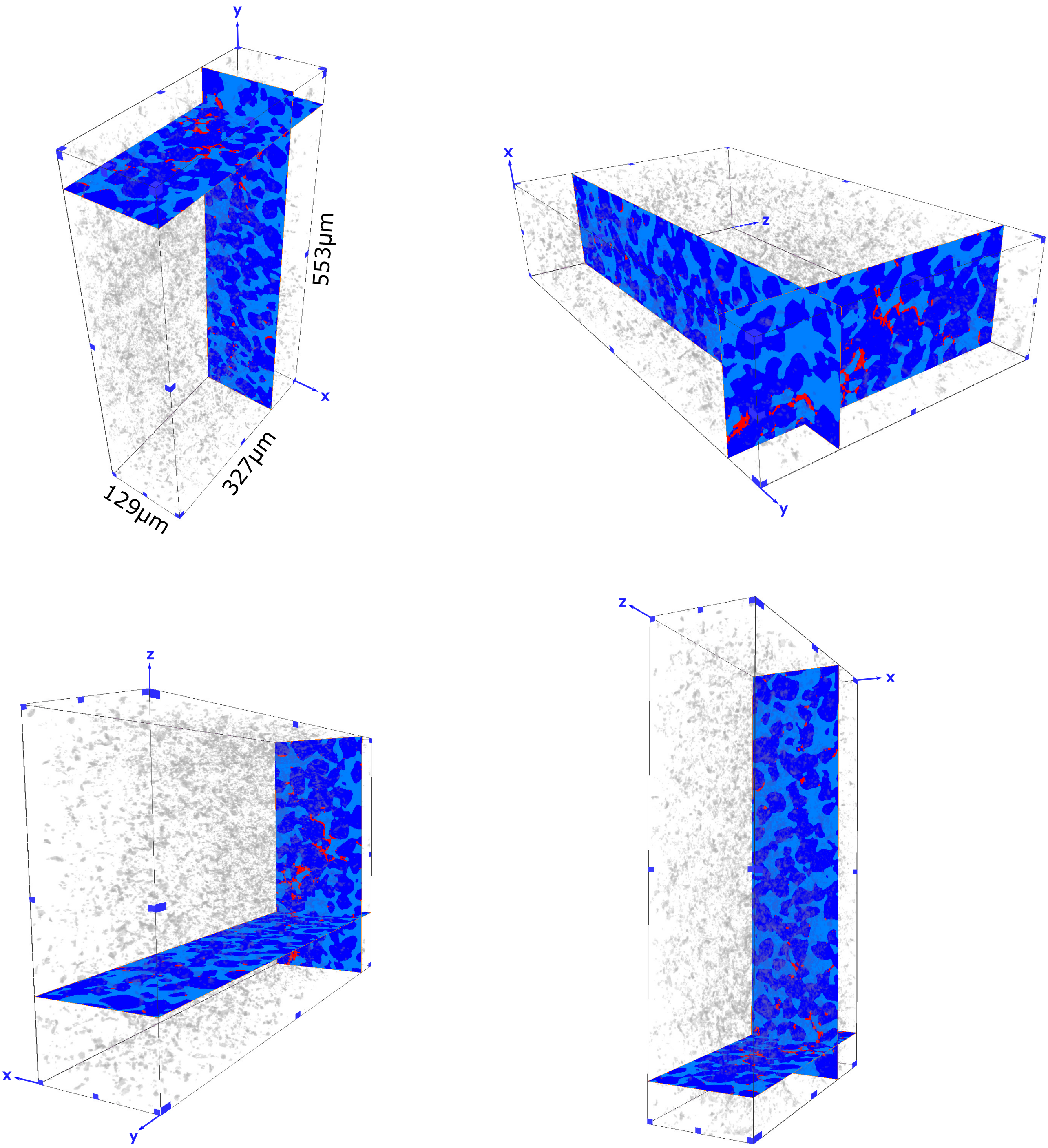

Fig. S19. Views of three-dimensional (3D) volume reconstructions of biomineralised hydrogel-sand scaffolds through X-ray μ-CT scans. Three-dimensional volume reconstruction (light blue; pores; dark blue, sand grains; pink and grey, $CaCO_3$ minerals).
